# Genetics of child aggression, a systematic review

**DOI:** 10.1038/s41398-024-02870-7

**Published:** 2024-06-11

**Authors:** Emiko Koyama, Tuana Kant, Atsushi Takata, James L. Kennedy, Clement C. Zai

**Affiliations:** 1 Tanenbaum Centre for Pharmacogenetics, Molecular Brain Science, Campbell Family Mental Health Research Institute, Centre for Addiction and Mental Health, Toronto, ON Canada; 2 Laboratory for Molecular Pathology of Psychiatric Disorders, RIKEN Center for Brain Science, Wako, Japan; 3 Department of Psychiatry, University of Toronto, Toronto, ON Canada; 4 Institute of Medical Science, University of Toronto, Toronto, ON Canada; 5 Laboratory Medicine and Pathobiology, University of Toronto, Toronto, ON Canada; 6 Stanley Center for Psychiatric Research, Broad Institute of MIT and Harvard, Cambridge, MA USA

**Keywords:** Genomics, Psychiatric disorders

## Abstract

Excessive and persistent aggressiveness is the most common behavioral problem that leads to psychiatric referrals among children. While half of the variance in childhood aggression is attributed to genetic factors, the biological mechanism and the interplay between genes and environment that results in aggression remains elusive. The purpose of this systematic review is to provide an overview of studies examining the genetics of childhood aggression irrespective of psychiatric diagnosis. PubMed, PsycINFO, and MEDLINE databases were searched using predefined search terms for aggression, genes and the specific age group. From the 652 initially yielded studies, eighty-seven studies were systematically extracted for full-text review and for further quality assessment analyses. Findings show that (i) investigation of candidate genes, especially of *MAOA* (17 studies), *DRD4* (13 studies), and *COMT* (12 studies) continue to dominate the field, although studies using other research designs and methods including genome-wide association and epigenetic studies are increasing, (ii) the published articles tend to be moderate in sizes, with variable methods of assessing aggressive behavior and inconsistent categorizations of tandem repeat variants, resulting in inconclusive findings of genetic main effects, gene-gene, and gene-environment interactions, (iii) the majority of studies are conducted on European, male-only or male-female mixed, participants. To our knowledge, this is the first study to systematically review the effects of genes on youth aggression. To understand the genetic underpinnings of childhood aggression, more research is required with larger, more diverse sample sets, consistent and reliable assessments and standardized definition of the aggression phenotypes. The search for the biological mechanisms underlying child aggression will also benefit from more varied research methods, including epigenetic studies, transcriptomic studies, gene system and genome-wide studies, longitudinal studies that track changes in risk/ameliorating factors and aggression-related outcomes, and studies examining causal mechanisms.

## Introduction

Childhood aggression is the most common reason for psychiatric referrals in children, comprising 64% of all referrals [1]. Youth are responsible for up to 200,000 homicides every year [2], and over 1000 children need emergency care for youth aggressive and physical assault-related injuries daily in the United States [3]. Aggression can be defined as behaviors that intend to create physical or emotional harm on another individual [4]. From an evolutionary standpoint, aggression has been seen as an advantageous adaptive strategy in obtaining and defending food and mates. Therefore, certain levels of aggression have continued to be positively selected for and maintained through the generations. Among preschool-aged children, temper tantrums can be considered normal [5], yet certain aggressive behaviors can develop into more severe pathological forms. Increased anger, irritation and frustration accompanied by persistent aggressive behaviors can have negative consequences throughout life such as peer rejection, relationship problems, poor academic performances and lower graduation rates, substance abuse issues, criminal behaviors, and financial and occupational difficulties [2, 3, 6–9]. Pathological aggressive behaviors not only lead to social and financial problems for the aggressor and the victims’ families, but can also have significant societal costs through increased needs for health and medical services, unemployment and welfare services, social services, and criminal justice services [3, 10, 11]. With a high prevalence of problematic aggressive behaviors, up to 30% of children in low income and single parent homes exhibit aggression [12–14]. Childhood aggression is a major public health concern that requires further understanding for better prevention and treatment strategies.

Although aggression can take on different forms and behaviors, researchers categorize aggression into two main categories: proactive and reactive aggression [15, 16]. Proactive aggression represents aggressive behaviors that are predatory and have premeditated purposes to harm others for external or internal personal gains [15]. On the other hand, reactive aggression is the reaction to a perceived threat [16, 17]. Proactive and reactive aggression are highly correlated and can co-occur or be expressed separately [15].

While maladaptive aggressive behaviors can exist without fitting into a specific diagnostic category, they can also be the core symptom of some of the psychiatric diagnoses such as oppositional defiant disorder (ODD), conduct disorder (CD), intermittent explosive disorder (IED) and antisocial personality disorder (ASPD) (For a review of disorders related to aggression, please see Blair et al. (2014) [18]). Symptoms of excessive aggressive behaviors start early and are highly stable [19], however expression of disruptive behaviors can change through development and can be different in children versus adults [18]. While persistent and extensive aggressive behaviors can be a symptom of ODD around age 12 [20], it can develop into CD during adolescence [21] and then further into ASPD in adulthood [18]. Therefore, it is crucial to assess aggressive behaviors from early childhood and adjust measurement methods and criteria based on the age and developmental stage of the patients. However, it is also important to note that aggressive behavior is not necessarily an essential symptom for these diagnoses.

Studies over two decades have demonstrated that there is a prominent genetic component to aggressive behaviors. It has been found that aggressive behaviors are highly heritable and genetic factors account for roughly 50–65% of the risk of high aggression [22, 23]. Initially, chromosomal abnormalities were studied in relation to aggressive behaviors. While XYY individuals have shown to have increased aggressive behaviors [24], they do not form the whole picture [25]. Therefore, there have been numerous studies analyzing the association between genes and aggression. One of the first and landmark studies that found the genetic contributions to aggressive behaviors is the study by Brunner et al. [26]. Brunner and colleagues investigated a family with a history of criminal behaviors and found that all males were lacking monoamine oxidase A enzyme activity, which encodes the monoamine oxidase A (MAOA) enzyme that regulates catecholamine and serotonin levels [27]. Following that, Caspi et al. (2002) [28] further investigated the association between *MAOA* genotypes and aggressive behaviors in abused males, further supporting that variants within genes may influence aggressive behaviors. There has been significant research conducted on the genetics of aggression in adults [29, 30]. Nonetheless, a study on two large population cohorts with ages from 12–73 years reported that effects of polygenic risk scores for childhood aggression appeared to decrease from childhood and adulthood to later life [31], suggesting that while child and adult aggression are genetically similar, it is conceivable that some genetic factors underlying ADHD in children and later life may be different [32], thus emphasizing the importance of studying the genetics of aggression in different age groups separately. There have been various genes in different biological pathways investigated in association to childhood aggression, including dopaminergic, serotonergic, vasopressin and oxytocin system genes (For an earlier review of the genetics of aggressive behaviors, please see Anholt & Mackat (2012) [33]). Researchers agree that childhood aggression is a polygenic trait, with numerous genes of small effects contributing to the phenotype.

Although numerous studies demonstrate evidence for genes underlying childhood-onset aggression and there are previous reviews focusing on certain genes, there has not been a review that systematically considers every gene that has been studied in relation to childhood aggression. The objective of this study was to systematically review the literature to provide a comprehensive summary and informed analyses of the genes influencing aggressive behaviors specifically in child and adolescent populations.

## Methods

### Literature search

The literature search was performed on May 20, 2022 using the PUBMED, MEDLINE, and PsycINFO databases. Pubmed search yielded 256 hits using the following search terms: ((aggression [MESH] OR aggressive behav* [TIAB] OR aggressive trait* [TIAB])AND (genes [MESH] OR genetics [TIAB] OR epigenetics [TIAB] OR genom* [TIAB] OR genot* [TIAB] OR GWAS [TIAB]) NOT (neoplasms [MESH] OR tumor* [TIAB] OR cancer* [TIAB])) and applying the filter for Child (birth to 18) and human studies. Ovid MEDLINE and PsycINFO searches yielded 111 and 280 articles respectively using the following search terms: ((aggression.mh. or aggressive behav*.ab. or aggressive behav*.ti. or aggressive trait*.ab. or aggressive trait*.ti.) AND (genes.mh. or genetics.ab. or genetics.ti. or epigenetics.ab. or epigenetics.ti. or genom*.ab. or genom*.ti. or genot*.ab. or genot*.ti. or GWAS.ab. or GWAS.ti.)); FILTER for “Child (0 to 18 years)” and (“human”). Five relevant articles were subsequently added as a result of manual search and articles available to the authors. Of the 652 hits, 215 articles were found to be duplicates and were removed, leaving 437 studies qualifying for initial screening. As a result of initial title and abstract screening, 137 articles were found to be irrelevant, leaving 300 articles eligible for full-text review.

### Inclusion and exclusion criteria

Since the purpose of our systematic review was to provide an overview of studies examining genes associated with childhood aggression, only articles examining aggression in children and adolescents aged 18 years or younger were included in this study. There were a few articles, however, that were included in our review despite the participants being older than 18 years of age at the time of assessment, because the participants were asked to rate their aggression retrospectively for when they were younger than age 18.

**Fig. 1:**
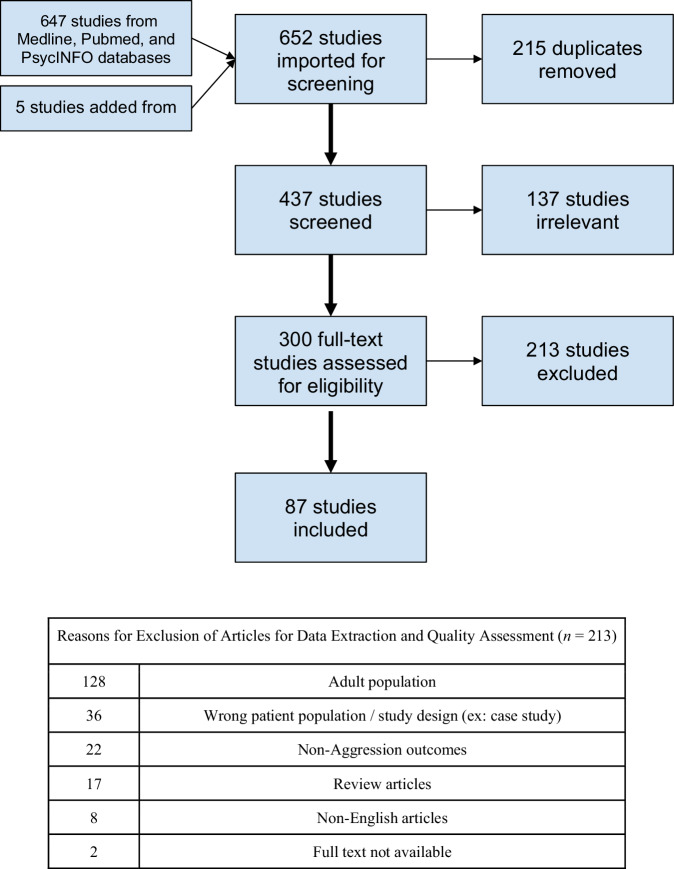
PRISMA flow chart. Flow chart showing the number of studies from our literature search and the number of studies removed during title/abstract screen and full-text review together a text box showing the reasons for exclusion.

Studies were excluded from further review if they were: (1) written in a language other than English, (2) dissertations & conference abstracts, (3) full text was not available, (4) review articles, (5) wrong patient population (ex: only studying children who were victims of aggression) or study design (ex:case studies), (6) included adult participants or (7) tested phenotypes other than aggression. As a result of these criteria, 212 studies were excluded. The most common reasons for exclusion were the inclusion of the adult population (*n* = 128), wrong patient population or study design (*n* = 36), and the outcome not being related to child aggression (*n* = 22). Eighty-seven articles were subject to data extraction and quality assessment. The PRISMA flow chart is shown in Fig. 1.

### Data extraction and quality assessment

Data extraction was performed by three independent reviewers (CZ, TK, and EK). The following information was extracted for each study: First author, year of publication, population characteristics, study type (twin/pedigree studies, longitudinal, candidate gene etc.), participants’ ancestry or country of origin, sex, age, sample size, genes assessed, assessment of aggression, and key findings related to aggression (Table 1). The quality of each article was evaluated regarding the risk of bias on a 4 point scale (ranging from low risk to critical) using the following criteria: sample size, confounding, participant selection, measurement of outcomes, selection of reported results and overall risk of bias (Table S1). Abstract screening, full text review, quality assessment, and data extraction were managed in Covidence.

**Table 1:** Summary of reviewed articles.

First author and year	Title	Population characteristics	Ancestry or Country	Sex	Age	Sample size	Study type	Genes assessed	Assessment of Aggression	Key findings (related to aggression)
Twitchell [34]	Serotonin transporter promoter polymorphism genotype is associated with behavioral disinhibition and negative affect in children of alcoholics.	Ongoing Michigan Longitudinal Study: Based on paternal antisocial and alcoholic behaviors, lower and lower-middle class families, without fetal alcohol syndrome	European	M; F	7–16: mean: 10.9, SD: 2.0	62 (45 M, 17 F)	Candidate gene	*5-HTTLPR*	CBCL	LL genotype: increased aggression (t = –2.35, df = 60, *p* < 0.05)
Lawson [43]	Association analysis of monoamine oxidase A and attention deficit hyperactivity disorder.	ADHD (diagnosed / suspected) children recruited from Child and Adolescent Psychiatry and Pediatric clinics in Greater Manchester, Cheshire, Bristol, Weston-Super-Mare and South Wales	UK, British Caucasian (parents and grandparents)	M; F	6 and 16 (mean 9.1, SD 1.9)	171	Candidate gene	*MAOA* rs6323 (T941G), VNTR (low activity = 3 R; high activity = 3.5, 4, 5 R)	Diagnosis of ADHD and conduct disorder and a broader definition of conduct disorder	NS rs6323 (case-control: OR = 0.77, 95%CI: 0.42–1.41; TDT: 23 informative transmissions, Chisq=0.39, df1, *p* = 0.53); VNTR low activity (3 repeats, OR = 2, 95% CI: 1.09–3.5, *p* = 0.025; TDT: 13 informative transmissions, Chisq=1.92, df1, *p* = 0.17); males only (3 repeats overtransmitted: Chisq=3, df1, *p* = 0.08)
Cadoret [90]	Associations of the serotonin transporter promoter polymorphism with aggressivity, attention deficit, and conduct disorder in an adoptee population.	Adoptees and adoptive parents from four different adoption agencies (starting from 1989)	US	M; F	2–6 & 6–12	87 (37 M, 50 F)	Candidate gene, adoption study	*5-HTTLPR*	Scale described by Loney et al (1980) with an additional 5 items	Main effect: NS preadolescent aggressivity (*p* = 0.09), significant adolescent aggressivity (*p* = 0.03) GxSex: preadolescent (*p* = 0.05) & adolescent (*p* = 0.03) aggression -> F: lower scores with S-allele; M: higher scores with S-allele Gx Biological parent alcoholism status: adolescent aggressivity (*p* = 0.03) -> increased score with S-allele with a genetic diathesis for alcoholism Gx Biological parent antisocial status: adolescent aggressivity (*p* = 0.01) -> increased scores with LL when antiosocial parent
Beitchman [45]	MAOA and persistent, pervasive childhood aggression.	Children with clinically high aggression and ethnically matched male adults with no history of psychiatric illnesses as non-aggressive controls - CAMP sample	80% European, 20% African-American	M	mean: 9.5, SD: 2.5	100 (50 case and 50 control)	Candidate gene, case-control	*MAOA* VNTR, rs6323 (low transcription = 3 R, high transcription = 4 R; 3.5 and 5 R excluded due to low frequency)	CBCL, TRF	4 R carriers -> increased aggression (*p* = 0.014)
Davidge [93]	Association of the serotonin transporter and 5HT1Dbeta receptor genes with extreme, persistent and pervasive aggressive behavior in children.	High aggression cases recruited via media advertisements and physician referrals; healthy adult controls	82% Caucasian, 10% African-American	M; F	6–16, mean: 10.08, SD: 2.53	50 high aggression children and locally recruited ethnically matched controls	Candidate gene	*5HT1Db* G861C (*HTR1B* rs6296), *5HTTLPR, 5HTT VNTR*	CBCL, TRF and 2 year history of aggression according to parents	Significantly reduced frequency of the *5HTT* VNTR 10 R allele in children displaying high-aggression phenotype compared with controls (*P* = 0.039), and 12 R allele more common in cases (*p* = 0.079). Probands also tended to have increased *5HT1Db* 861 C allele frequency, but N.S. *5HTTLPR* not significant.
Bearden [62]	Effects of COMT genotype on behavioral symptomatology in the 22q11.2 deletion syndrome	22q11.2 Deletion Syndrome	92% Caucasian, Philadelphia USA	M; F	Mean: 10.9 Met: 10.1, SD: 2.4; Val: 11.5, SD: 3.7	38 (61% F)	Candidate gene	*COMT* rs4680 (Val158Met)	CBCL	NS association between Val/Met genotype and aggressive scores
DeYoung [81]	The dopamine D4 receptor gene and moderation of the association between externalizing behavior and IQ.	(a) CAMP, (b) Adults with ADHD, (c) Longitudinal study (Montreal)	(a) 80% Caucasian, (b) 98% Caucasian (c) French-speaking Canadian	M	(a) 5–15 (mean, 9.89 SD: 2.53 years); (b) 18-56 (mean 35.17, SD:10.22 years); (c) longitudinal sample from 6, 10, 11, 12 years	(a) 50 (b) 67 (c) 87	Candidate gene	*DRD4* exon3 VNTR	(a) CBCL, TRF parent ratings of psychopathy; (b) Spouse or mother ratings of anger control; (c) Teacher ratings of aggression, opposition	NS main effect; Males with no 7 R: externalizing behavior negatively correlated with IQ (mean r = 0.43, *p* < 0.001). Males with at least 1 copy of 7 R: externalizing behavior and IQ not correlated.
van Donkelaar [111]	Pleiotropic contribution of MECOM and AVPR1A to aggression and subcortical brain volumes.	EAGLE (early genetics and Lifecourse Epidemiology)Consortium	European	M; F	mean: 8.44, SD:4.16	18988 (EAGLE)	Genome-wide gene-based cross-trait meta-analyses	N/A	EAGLE - CBCL, SDQ; BIG - Reactive-Proactive Questionnaire	Aggression: *MECOM* (MDS1 and EVI1 complex gene) (*p* = 1.67e–06) and *AVPR1A* (*p* = 3.40e–05)
Beitchman [86]	Serotonin transporter polymorphisms and persistent, pervasive childhood aggression.	Children with clinically high aggression - CAMP sample	Canada	M; F	5–15; mean:9.54, SD: 2.62	154 (77 cases, 77 controls)	Candidate gene, case-control	5*-HTTLPR, 5-HTT* VNTR	CBCL and TRF	Childhood aggression significantly associated with “low expressing” genotypic variants of the 5-*HTTLPR* polymorphism (S/S, Lg/S, Lg/Lg) (N = 77, *p* = 0.049, OR = 2.37, CI = 1.10–5.08) and the S/S genotype alone (*p* = 0.047, OR = 2.65,CI = 1.12–6.28)
Haberstick [87]	Family-based association test of the 5HTTLPR and aggressive behavior in a general population sample of children.	Twins and parents from Longitudinal Twin Study	USA; >90% self-report Caucasian, 10% African American; Hispanic American; Asian American; Mixed	M; F	Annually 7–12	366 families (327 mothers, 128 fathers, and 732 individual twins (174 MZ M, 208 MZ F, 184 DZ M, 166 DZ F))	Candidate gene, twin, longitudinal	*5HTTLPR*	CBCL, TRF	N.S. *HTTLPR* for parental ratings; S-allele associated with TRF aggressive behavior at age 9 for within family test (chisq=4.34, *p* = 0.04)
Bakermans-Kranenburg [83]	Gene-environment interaction of the dopamine D4 receptor (DRD4) and observed maternal insensitivity predicting externalizing behavior in preschoolers.	Netherlands, Twin Registar - Twin (only one sibling was chosen for this study)	Netherlands	M; F	10 months of age	47 (23 M, 24 F)	Candidate gene	*DRD4* exon3 VNTR	CBCL	NS main effect GxE: *DRD4* x maternal sensitivity interaction (*p* = 0.02): 7-repeat + insensitive mothers -> increased aggression
Nobile [77]	Socioeconomic status mediates the genetic contribution of the dopamine receptor D4 and serotonin transporter linked promoter region polymorphisms to externalization in preadolescence.	Italian Project on Preadolescent Mental Health (Progetto Italiano Salute Mentale Adolescenti or PrISMA) and Ponte Lambro (PL) inhabitants	96.4% European	M; F	10–14	422 + 165 = 607 (309 M, 298 F); 589 successfully genotyped	Candidate gene, GxE	*DRD4* exon3 VNTR, *5-HTTLPR*	CBCL	Aggression: *DRD4* (F = 9.36, df=1,579, *p* = 0.002) *5-HTTLPR* (F = 4.87, df=1,579, *p* = 0.028) *DRD4x5-HTTLPR* (F = 11.09, df=1,579, p = 0.001) *DRD4*xSES (F = 9.22, df=1,579, p = 0.002) *5-HTTLPR*xSES (F = 5.06, df=1,579, *p* = 0.025), Increased aggresssion with: LL 5-*HTTLPR* genotype, carrying 1+ long (6–8 R) copies of the *DRD4* exon-3 polymorphism, in families with low SES
Caspi [60]	A replicated molecular genetic basis for subtyping antisocial behavior in children with attention-deficit/hyperactivity disorder.	(a) Cardiff Clinical sample (100% ADHD) from child psychiatry and child health clinics and 2 birth cohort studies (b) E-RISK (8% ADHD), (c) Dunedin (6% ADHD)	(a) 100% UK White origin, (b) 100% England, Wales (c) 100% New Zealander	M; F	(a): 5.4 years old (mean: 9.25) (b) 5 and 7 years olds (c) 11, 13 and 15 years olds	(a) 241; (b) 1116 families with same-sex twins; (c) 1037	Candidate gene	*COMT* rs4680 (Val158Met)	(a)Child and Adolescent Psychiatric Assessment (DSM-IV) -- parent version; (b) CBCL, TRF @ 7 years (c) composite index of antisocial behavior in adolescence and in adulthood	Among children with ADHD, Val/Val genotype carriers had higher aggression than Met carriers (a, b,c). No significant association among non-ADHD children (b, c).
Oades 2008 [80]	The influence of serotonin- and other genes on impulsive behavioral aggression and cognitive impulsivity in children with attention-deficit/hyperactivity disorder (ADHD): Findings from a family-based association test (FBAT) analysis.	International Multicenter ADHD Genetics (IMAGE) study: ADHD cases and siblings without the disorder	European	M; F	5–7: mean:10.9	1180 children (793 M, 387 F) - 607 families, 603 affected with ADHD-combined type; 1116 families successfully genotyped	Candidate gene, family-based test	14 genes with 582 SNPs: *HTR1A, HTR1B, HTR1E, HTR2A, HTR3B, TPH2, SERT/SLC6A4, DRD1, DRD4, DAT1/SLC6A3, BDNF, NURR-1, FADS2, PNMT*	CPRS, CTRS, SDQ	Overt aggression: 6 SNPs from *BDNF, DRD4, HTR1E, PNMT*, and *TPH2* (*p* < =0.05) Aggressive behavioral impulsivity: *DRD4* variant - exon 3: 3 repeats (*p* = 0.014)
Shleptsova 2008 [154]	Role of renin-angiotensin system in the formation of emotional state in humans.	Athletes	Russia	M; F	14 ± 3 (female athletes) and 24 ± 5 years (male athletes) 17 ± 4 years (volunteers)	189 athletes (130 M, 59 F) and 212 volunteer controls (45 M, 167 F)	Candidate gene	Angiotensin-converting enzyme (*ACE*), I/D	Bass—Darky Questionnaire	Physical aggression in I/I genotype carriers lower than D allele carriers among female synchronized swimmers for age 10–14 (*p* = 0.05) and >=15 (*p* = 0.03).
Weder [49]	MAOA genotype, maltreatment, and aggressive behavior: the changing impact of genotype at varying levels of trauma.	Children removed from parental care within the past 6 months due to reports of abuse or neglect (or both), and 41 community control subjects	24% European American, 25% Hispanic, 31% African American, 19% biracial	M; F	5–15	114 (73 cases, 41 control)	candidate gene, GxE	*MAOA* uVNTR (low activity = 2,3,5 R; high activity = 3.5, 4 R)	TRF	Significant interaction between moderate trauma and low activity (2, 3, and 5 repeat) *MAOA* genotype. Extreme levels of trauma, associated with high aggression irrespective of genotype.
Hohmann [78]	Evidence for epistasis between the 5-HTTLPR and the dopamine D4 receptor polymorphisms in externalizing behavior among 15-year-olds.	Mannheim Study of Children at Risk	Caucasian	M; F	15	298 (144 M, 154 F)	Candidate gene, GxG	*DRD4* exon 3 VNTR, *5-HTTLPR*	YSR, CBCL (primary caregiver), TRF (teacher)	Significant main effect of *DRD4*: 7 repeats associated with increased aggressive behavior (*p* = 0.01) NS: 5-*HTTLPR* main effect Significant GxG interaction: Highest aggression among carriers of homozygous 5-*HTTLPR* short allele and *DRD4* 7 repeat (*p* = 0.002)
Sysoeva [88]	Aggression and 5HTT polymorphism in females: study of synchronized swimming and control groups.	Synchronized swimmers from the Moscow Professional Sport Club and non-athlete controls	Caucasian	F	Swimmers 10–18 (mean: 13 ±+ 0.3), control: 10–18 (mean: 13 ± 0.3)	62 synchronized swimmers and 64 age-matched control	Candidate gene	*5-HTTLPR*	Buss-Durkee Hostility Inventory adapted for Russian population and 10-26 y/o	The “covert aggression” scale showed significant effect of 5-*HTT* polymorphism, F(2, 159) = 6.32, *p* = 0.002. (SS polymorphism associated with higher scores on covert aggression, indirect hostility; lower scores on negativism) When the older control group (20 years old) was excluded from the analysis, the effect of 5-*HTT*, F(2,119) = 8.63, *p* = 0.0003, remained significant.
DiLalla [85]	Genetic and gene-environment interaction effects on preschoolers’ social behaviors.	Southern Illinois Twins and Siblings Study	97% Caucasian, 3% Latino	M; F	3–5	62 unrelated children (28 M, 34 F) selected from 129 twin and triplet children	Candidate genes, longitudinal, GxE	*DRD4* exon 3 VNTR	Parent-twin triad interactions at age 3 years, CBCL, Behavioral Styles Questionnaire, Peer-play interactions at age 5	Model with genotype and parental sensitivity as predictors significant,F(2,49) = 4.35, *p* < 0.05. Children with *DRD4*-L significantly more aggressive in a low-aggression environment. Children with *DRD4*-L who had insensitive parents were more likely to have externalizing problems. Children with *DRD4*-L less likely to share with one other and parents were less sensitive during parent-twin triadic interactions. Genotype interacted with peer aggression to affect child aggression during peer play at age 5.
Albaugh [61]	COMT Val158Met genotype as a risk factor for problem behaviors in youth.	Vermont Family Study	USA	M; F	6–18, mean 10.99; SD 3.66	149	Candidate gene	*COMT* rs4680 (Val158Met)	CBCL	AGG raw score on average 2.9 points higher in Met-carriers than ValVal (beta=0.146, SE = 0.06, *p* = 0.016), controlling for covariates. On average 1 point higher for direct aggression score (beta=0.178, SE = 0.065, *p* = 0.007) and on average 1.9 points higher for relational aggression score (0.123, SE = 0.06, *p* = 0.041).
Edwards [52]	MAOA-uVNTR and early physical discipline interact to influence delinquent behavior.	Child Development Project (CDP) -- children recruited during pre-registration for kindergarten in 1987 and 1988	Nashville, TN; Knoxville, TN; and Bloomington, IN; 81% European American, 17% African American, 2% other ethnic groups- European American	M	Longitudinal from around age 5	186	Candidate gene	*MAOA*-uVNTR (low=2, 3, 5 R, high=3.5, 4 R)	CBCL (age 6–17), YSR (12, 14–17, 19–22), TRF (6–13)	NS main effects of genotypes on externalizing, aggression, or delinquency reported by either mothers or teachers; NS for self-reports
Kiive [155]	Effect of alpha2A-adrenoceptor C-1291G genotype and maltreatment on hyperactivity and inattention in adolescents.	European Youth Heart Study (1998/99); later incorporated into the longitudinal Estonian Children Personality Behavior and Health Study	Estonia	M; F	mean: 15.3, SD: 0.5	429 (196 M, 233 F)	Candidate gene	*ADRA2A* C1291G (rs1800544)	Teacher report - 7-point Hyperactivity Scale of af Klinteberg (1988)	NS genetic main effect; NS GxE with maltreatment (maltx) for males; Females with CC genotype and high maltx had higher aggression than females with GG and high maltx (*p* = 0.03), and females with GG and high maltx had lower aggression scores than those with GG and low maltx score (*p* = 0.005).
Dick [156]	CHRM2, parental monitoring, and adolescent externalizing behavior: evidence for gene-environment interaction.	Child Development Project (CDP): Community sample	US: 81% European American, 17% African American, 2% others	M; F	Longitudinal: 10–17 annual data collection for 5+ years	452 (93% of original Child Development Project sample)	Candidate gene, GxE, longitudinal	*CHRM2* (9 SNPs)	CBCL, YSR	NS main effect GxE: 3 *CHRM2* SNPs (rs36210735, rs7800170, rs8191992) x parental monitoring (*p* = 0.017–0.045) -> difference between genotypes decreased as parental monitoring increased; TT, CC, TT highest when low in parental monitoring, and lowest when high in parental monitoring, respectively Replication sample: TRacking Adolescents’ IndividualLives Survey (TRAILS), ages 10-18, Dutch -> GxE with rs10271552 (*p* = 0.035)
Thibodeau [157]	Child maltreatment, impulsivity, and antisocial behavior in African American children: Moderation effects from a cumulative dopaminergic gene index.	Low income children w/wo maltreatment	African American	M; F	mean: 10.07, SD: 1.6 (6–13)	1012; Maltreated (*n* = 493) and nonmaltreated children (*n* = 519) (500 F)	Gene index - polygenic index	*DRD4* exon3 VNTR, *DAT1* VNTR and five genotypes in *DRD4, DRD2, DAT1, COMT*	Peer rating, TRF, California Child Q-set (for impulsivity)	Impulsivity partially mediated maltreatment subtypes and antisocial behavior (β = 0.173, *p* < 0.001), and number of differentiating dopaminergic genotypes moderated these relations -- GxE (b = 0.016, *p* = 0013). The more differentiating genotypes the stronger the relationship between maltreatment and impulsivity, thus a stronger mediational effect of impulsivity on antisocial behavior.
Zai [99]	Dopaminergic system genes in childhood aggression: Possible role for DRD2.	CAMP - clinically aggressive children and adult controls	77.6% Caucasian, 5.6% African-Canadian, 16.7% mixed	M; F	5–16, mean: 10.8, SD: 3.07	104 M and 40 F, + 144 adult controls	Candidate gene, case-control	*DRD2* (5 SNPs), *DRD4* exon3 VNTR, *DAT1* VNTR	CBCL, TRF, 2+ years history of aggression	Aggressive children significantly more likely to have at least one copy of *DRD2* A-241G G allele (genotypic *p* = 0.02; allelic *p* = 0.01). *DRD2* rs1079598 CC genotype overrepresented in aggressive children compared to controls (*p* = 0.04). *DRD2* TaqIA T allele (p = 0.01) and the TT genotype (*p* = 0.01) significantly overrepresented in aggressive children.
Lacourse [41]	A longitudinal twin study of physical aggression during early childhood: evidence for a developmentally dynamic genome.	Longitudinal Quebec Newborn Twin Study	Quebec, Canada	M; F	20, 32, and 50 months	667 twin pairs (254 MZ, 413 DZ pairs)	Twin, longitudinal, heritability	N/A	3 items mother report - how many times child hits, bites, kicks; fights; and attacks another (0 = never, 1 = sometimes, 2 = often)	Initial genetic contribution substantially decreased over time, while new genetic effects appeared later on. Individual differences in initial level (ai=0.79, 95% CI 0.69–0.88), and change rate (as=0.29, 95% CI 0.11–0.39) explained by genetic factors. Stability of PA explained by genetic factors. Non-shared and shared environments no effect on the stability, initial status and growth in PA.
Beitchman [158]	Childhood aggression, callous-unemotional traits and oxytocin genes.	clinically aggressive children and ethnically and gender matched adult controls -- CAMP sample	84% Caucasian, 4.9% African-Canadian, 11.1% others	M; F	Cases: 6-16, mean: 11.81, SD: 2.85; controls: mean: 25.64, SD: 8.72 years	162 (106 M, 56 F)	Candidate gene, case-control	*OXT* (3SNPs), *OXTR* (3SNPs)	DISC Checklist to primary caretaker, review of participants’ health records; CDI, Psychopathy Screening Device; CBCL, TRF, 2+ years of aggression according to parent	NS *OXT* and *OXTR* in case-control analysis
Zai [99]	Possible genetic association between vasopressin receptor 1B and child aggression.	Child aggression cases and non-aggressive adult controls matched by sex and ethnicity -- CAMP sample	European	M; F	mean: 11.73, SD: 2.88	177 high aggression cases (59 F) and 177 sex and ethnicity matched adult controls	Candidate genes	*AVP* (5SNPs), *AVPR1A* (2SNPs), *AVPR1B* (4 SNPs)	CBCL and TRF; 2+ years of aggression according to parent	*AVPR1B_*rs35369693 C allele less common in the aggressive child cases than in the healthy controls (ORC = 0.37, 95% confidence interval: 0.17–0.77; *P* = 0.009). Results remained significant with sex and ethnicity as covariates (*P* = 0.007) or in Europeans-only analysis (ORC = 0.43; 95% CI: 0.21–0.9; *P* = 0.024). *AVPR1B*_rs35369693-rs2867650 C–A haplotype being under-represented in aggressive children (P-window=0.003; ORC-A = 0.28; 95% confidence interval: 0.13–0.64; haplotype-specific *P* = 0.001). *AVPR1B*_rs28375468-rs35369693 G-C haplotype underrepresented in aggressive children (P-window=0.038; ORG-C = 0.39; 95% CI: 0.18-0.84; haplotype-specific *P* = 0.013). Two-marker haplotypes associated with *AVPR1B*_rs35369693 nominally significant in European-only analysis, with the *AVPR1B*_rs35369693-rs28676508 C–A haplotype being under-represented in child cases (haplotype window *P* = 0.010; PC–A = 0.003).Markers in *AVP* and *AVPR1A* genes not associated with child aggression.
Malik [159]	The role of oxytocin and oxytocin receptor gene variants in childhood-onset aggression.	Clinically aggressive children and adult controls -- CAMP sample	82% Caucasian, 8% African-Canadian and 10% other and mixed ethnicities	M; F	6–16 (11.45 ± 3.04 years); controls (25.87 ± 8.99 years)	236 (162 M, 74 F); 160 adult controls (106 M, 54 F)	Candidate gene, case control	*OXT* (3 SNPs), *OXTR* (5 SNPs)	CBCL, TRF, 2+ years history of aggression	C allele for *OXTR* rs1042778 was over-represented in the male aggressive cases (*P* = 0.016) compared to male adult controls; haplotype consisting of *OXTR* rs1042778 allele C and *OXTR* rs6770632 allele G was over-represented in male aggressive cases vs. controls (window *P* = 0.032, specific *P* = 0.0024, OR = 1.9); haplotype consisting of *OXTR* rs1042778 allele C and *OXTR* rs53576 allele T was over-represented in male aggressive cases vs. controls (window *P* = 0.036, specific *P* = 0.016, OR = 2.5); similar findings in Caucasian subsample
Pingault [46]	Age-dependent effect of the MAOA gene on childhood physical aggression.	Longitudinal study of kindergarten children in Quebec, Canada	European	M	Longitudinal: 6–12	436	Candidate gene, longitudinal	*MAOA* (5 SNPs)	Social Behavior Questionnaire - teacher rating	Slope of physical aggression: rs5906957 main effect (*p* = 0.04): levels of aggression for T carriers decreased less (–0.01 each year) than C carriers (–0.08 each year) Lower initial levels of physical aggression with T than C: rs5906957 (*p* = 0.05) Similar results for rs5953385 and rs2283725 (*p* < 0.05)
Hirata [63]	Study of the catechol-O-methyltransferase (COMT) gene with high aggression in children.	Children clinically- referred for behavioral problems and persistent aggression & healthy adult controls - CAMP sample	Canada; 77.6% European Caucasian, 5.6% African Canadian, 16.7% others	M; F	6–16 (mean:10.8, SD:3.07)	104 M & 40 F with healthy adult controls matched based on ethnicity and gender	Candidate gene, case-control	*COMT* (4 SNPs)	CBCL, PSD, TRF, 2+ year history of aggresion	Significant rs6269 (*p* = 0.019), and trend for rs4818 (*p* = 0.064)
Pickles [53]	Evidence for interplay between genes and parenting on infant temperament in the first year of life: monoamine oxidase A polymorphism moderates effects of maternal sensitivity on infant anger proneness.	Wirral Child Health and Development Study: stratified epidemiological cohort recruited during pregnancy	UK	M; F	29 weeks, 14 months	193 (92 M, 101 F)	Candidate gene, GxE, longitudunal observational study	*MAOA* VNTR (low activity = 3, 5 R; high activity = 3.5, 4 R)	29 weeks + 14 months: Infant Behavioral Questionnaire - Revised	GxE: -> *MAOA* 3.5 & 4 R + decreased maternal sensitivty-> increased anger (*p* = 0.003), when adjusted for confounders (*p* = 0.0001)
Hakulinen [94]	Serotonin receptor 1B genotype and hostility, anger and aggressive behavior through the lifespan: the Young Finns study.	Finn (Cardiovascular Risk in Young Finns study)	Finnish	M; F	3–12, mean: 9.06, SD: 2.4	967 (448 M, 519 F)	Candidate gene, longitudinal	*HTR1B* rs6296	Three item yes/no question regarding aggressive behavior at first assessment. Four item, five point scale question regarding aggressive behavior at 2nd assessment.	Individuals with C/C more aggressive than C/G (ß=-0.34, *p* = 0.008) and G/G (ß=–0.36, *p* = 0.004); rs6296 modified the association between childhood aggressive behavior and adult hostility.
Farbiash [79]	Prediction of preschool aggression from DRD4 risk, parental ADHD symptoms, and home chaos.	Ben-Gurion University Infant Developmental Study (BIDS) -- longitudinal study of children born to fathers with ADHD symptoms	Israel	M	4.5 years, mean: 4.44, SD: 0.16	84	Candidate gene, GxE	*DRD4* exon 3 VNTR	CBCL - 19-item Aggressive Behavior subscale (sum of mother and father ratings)	*DRD4* 7 R associated with significantly higher aggression than those without 7 R allele (beta=0.21, *p* < 0.05)
Malik [100]	The role of genetic variants in genes regulating the oxytocin-vasopressin neurohumoral system in childhood-onset aggression.	Children with clinically high aggression - CAMP sample	Caucasian	M; F	6–16—cases: mean: 11.92 (SD: 2.89); controls: mean: 11.31 (SD: 2.56)	182 cases and 182 controls	Candidate gene, case-control	*OXT* (6 SNPs), *OXTR* (9 SNPs), *AVP* (5 SNPs), *AVPR1A* (2SNPs), *AVPR1B* (4SNPs), *CD38* (2SNPs)	CBCL, TRF (YSR for some controls)	*OXTR* rs237898 A-allele over-represented in cases (*p* = 0.018); haplotype consisting of *OXTR* rs237898A and rs237902C was over-represented in cases (window *p* = 0.032, haplotype-specific *p* = 0.010); In males: rs237898 A allele (*p* = 0.006) and the AA genotype over-represented in cases (*p* = 0.033); *OXTR* rs237902 C allele (*p* = 0.007) and CC genotype (*p* = 0.021) over-represented in male cases; *OXTR* rs237898A and rs237902C haplotype over-represented in male cases (window *p* = 0.025, haplotype-specific *p* = 0.021); In females: *OXTR* rs6770632 T allele over-represented in cases (*p* = 0.028) and rs1042778 AA genotype over-represented in cases (*p* = 0.048); Haplotype rs6770632T-rs1042778A over-represented in female cases (window *p* = 0.014, haplotype-specific *p* = 0.081); For *AVP* rs3761249: A allele over-represented (*p* = 0.008) and AA genotype (*p* = 0.037) was under-represented in cases, and rs2282018G-rs3761249C haplotype over-represented in male cases (window *p* = 0.012, haplotype-specific *p* = 0.011), *AVP* rs1410713T-rs2740204T haplotype over-represented in male cases (window *P* = 0.035, haplotype-specific *p* = 0.013); In females: *AVPR1A* rs11174811 G allele was over-represented in female cases (*p* = 0.04)
Villafuerte [160]	Genetic variation in GABRA2 moderates peer influence on externalizing behavior in adolescents.	Ongoing Michigan Longitudinal Study: one parent with lifetime alcohol dependence /abuse diagnosis	US: 95% European American, 2.5% White Hispanic; 2.5% African American or biracial	M; F	15–17	244 (169 M, 75 F)	Candidate gene, GxE	*GABRA2* rs279826	TRF	NS main effect NS GxE on aggression
Zohsel [84]	Mothers’ prenatal stress and their children’s antisocial outcomes--a moderating role for the dopamine D4 receptor (DRD4) gene.	Ongoing epidemiological cohort study	99% European descent, Germany	M; F	Longitudinal: 8, 11, 15	308 (150 M, 158 F)	Candidate gene, GxE, longitudinal	*DRD4* exon3 VNTR	8, 11, 15 years: CBCL, before 15: the Mannheim Parent Interview, 15: Schedule for Affective Disorders and Schizophrenia in School Age Children K-SADS	GxE: Homozygous 7 R carriers + increased prenatal stress -> increased externalizing behaviors Homozygous 4 R carriers -> insensitive to prenatal stress
Kiive [48]	Mitigating aggressiveness through education? The monoamine oxidase A genotype and mental health in general population.	Estonian Children Personality, Behavior and Health Study	Estonian	M; F	mean: 15.6, SD: 0.6 at original sampling	593 (260 M, 333 F)	Candidate gene, longitudinal	*MAOA* VNTR (low activity = 2, 3, 5 R; high activity = 3.5, 4 R)	Teacher report - 7-point Hyperactivity Scale and Swanson, Nolan and Pelham Questionnaire IV (SNAP-IV)	NS association between *MAOA*-uVNTR and aggression
Hygen [65]	Catechol-O-methyltransferase Val158Met genotype moderates the effect of disorganized attachment on social development in young children.	Trondheim Early Secure Study -- Birth cohorts and their parents	Norwegian	M; F	T1 - 54.79 months, T2 - 80.52 months	704 (647 (355 M) in T2)	Candidate gene	*COMT* rs4680 (Val158Met)	CBCL, TRF	High disorganization + ValVal -> greater increase in aggression over time High disorganization + MetMet -> significant decrease in aggression over time
Hamshere [110]	High loading of polygenic risk for ADHD in children with comorbid aggression.	Case: Cardiff sample - from mental health services or community pediatric outpatient clinics in UK Comparison: Wellcome Trust Case Control Consortium (Phase 2)	UK	M; F	Cardiff: 6-17 years (mean:10.7, SD:2.8)	452 case subjects (M = 389), 5,081 comparison subjects	GWAS, polygenic analysis	N/A	ADHD & Conduct - Child and Adolescent Psychiatric Assessment	ADHD polygenic risk score increased with total conduct disorder score (b = 0.118, t = 2.530, *p* = 0.006), polygenic risk score also increased with number of aggressive conduct disorder symptoms (b = 0.151, t = 3.152, *p* = 0.002)
Hygen [66]	Child exposure to serious life events, COMT, and aggression: Testing differential susceptibility theory.	Trondheim Early Secure Study - Birth cohort (2003 and 2005)	95.5% Norwegian	M; F	4–5.58, mean: 4.56	704 (355 M)	Candidate gene, GxE	*COMT* rs4680 (Val158Met)	TRF	No main effects of *COMT* and serious life events on aggression. Significant interactive effect of childhood serious life events and *COMT* genotype: Children with many serious life events and were Val homozygotes exhibited more aggression (*p* = 0.02) compared to Met-carriers. Without serious life events, Val homozygotes displayed significantly lower aggression scores than Met carriers (*p* = 0.03).
Feinberg [38]	Parenting and adolescent antisocial behavior and depression: evidence of genotype x parenting environment interaction.	Non-shared Environment in Adolescent Development Project (1st wave)	U.S., 94% of mothers and 93% of fathers European American	M; F	9–18	720 families with at least 2 children (93 with MZ twins, 99 DZ or unknown, 95 full siblings from nondivorced families, 182 full siblings, 109 half-siblings, 130 unrelated siblings)	Twin / pedigree	N/A	Behavior Problems Index (6 items on delinquency and 4 items on aggression), CBCL, 9-item subscale from Behavior Events Inventory	Genetic factors (A), shared environment (C), nonshared environment (E) account for 45%, 33%, 22% of variance in antisocial behavior in families with low parental negativity
Provençal [126]	Association of childhood chronic physical aggression with a DNA methylation signature in adult human T cells.	Children from low socioeconomic status families; high aggression cases and control without history of high physical aggression	Caucasian	M	longitudinal 6-15 years	20 (8 chronic physical aggression and 12 controls)	Epigenetics, case-control	T cells DNA methylation	Cases had history of aggression (unclear how it was determined) from 6-15 years	448 distinct gene promoters differentially methylated in CPA. Of these promoters, 277 more methylated in the control group, and 171 more methylated in the CPA group. Significant overrepresentations in chromosomes 4 and 5 (*p* = 0.01). Five genes previously linked with aggression were differentially methylated (*AVPR1A, HTR1D, GRM*5 less methylated in CPA group, *DRD1*, *SLC6A3* more methylated in CPA group).
Salvatore [161]	Intergenerational continuity in parents’ and adolescents’ externalizing problems: The role of life events and their interaction with GABRA2.	(a) Child Development Project & (b) FinnTwin12	European American (a)	M; F	12–17 (a), 14 (b)	(a) 324 (b) 802	Candidate gene (a,b), longitudunal (a)	*GABRA2* rs279871	CBCL and TRF (a), Behavioral Problems scale from the Teacher Form of the Multidimensional Peer Nomination Inventory (b)	CDP (a): GxE: no G allele + life events -> increased externalizing (*p* = <0.05) FinnTwin 12 (b): NS parallel moderation trends (*p* = <0.11)
Ma [44]	Electrophysiological responses of feedback processing are modulated by MAOA genotype in healthy male adolescents.	Chinese adolescents -- class rosters by random number table method in a regular senior high school in Changsha.	Han Chinese	M	14–17, mean:15.96, SD:0.97	72	Candidate gene	*MAOA* VTNR (low = 2,3,5 R; high = 3.5, 4 R)	Self-reported Chinese version of Buss-Perry Aggression Questionnaire; Simple monetary gambling task	*MAOA*-L associated with higher aggression, which was inversely correlated with dFRN across two groups. Compared with *MAOA*-H group, BPAQ-C total scores in *MAOA*-L group were significantly higher [t(70) = 2.630, *p* = 0.010], similarly observed both the anger and hostility subscales [anger: t(70) = 2.150, *p* = 0.035; hostility: t(70) = 2.048, *p* = 0.044].
Elam [115]	Gene set enrichment analysis to create polygenic scores: a developmental examination of aggression.	Low-income families with 2-year-old children - Women, Infants, and Children Nutritional Supplement Programs (WIC)	US, 13% Latino, 28% African American, 50% EuropeanAmerican, 13% biracial, and 9% other groups (e.g., NativeAmerican, Asian American)	M; F	2–14 - early childhood (2–5 years old), and middle childhood (7.5–10.5 years old)	515 (49% F)	PRS, longitudunal	N/A	CBCL	The functional-PRS (SNPs from gene sets and with known biological function) was associated with aggression in both early and middle childhood. The all-PRS and mapped-PRS (from SNPs mapped to gene sets using GSEA) were not significantly associated with aggression at any age.
van Goozen [162]	Identifying mechanisms that underlie links between COMT genotype and aggression in male adolescents with ADHD.	DSM-IV ADHD or ICD-10 Hyperkinetic Disorder	UK community clinics	M	10–17, mean: 13.95	194	Candidate gene	*COMT* rs4680 (Val158Met)	Development and Well Being Assessment	Mediation analyses showed that the bias corrected confidence intervals for the path coefficients did not cross zero for both models (0.091–0.613 for fear conditioning; 0.095–0.288 for fear empathy), consistent with a significant indirect effect of *COMT* genotype on aggression; Table S2: Aggression scores: VV: mean1.1 SD1.3, VM: mean1.1 SD1.2, MM: mean1.4 SD1.7, *p* = 0.57
Zhang [50]	The interactive effect of the MAOA-VNTR genotype and childhood abuse on aggressive behaviors in Chinese male adolescents.	Healthy students from 4 middle schools from Changsha, Hunan, China	China	M	mean:15.81, SD:1.70	507	Candidate gene, GxE	*MAOA* uVNTR (low activity = 3, 5 R; high activity = 3.5, 4 R)	YSR	NS main effect of *MAOA* VNTR; significant interaction MAOA x total abuse (β = −0.258, t = −2.687, *P* = 0.008): Among those with childhood maltreatment, L (3 or 5 repeats) group associated with higher aggression scores vs H (3.5 or 4 repeats) group; also *MAOA* x physical abuse (β = −0.273, t = −2.114, *P* = 0.035) and MAOA x emotional abuse (β = −0.255, t = −2.240, *P* = 0.026)
Chao [119]	The causal role of alcohol use in adolescent externalizing and internalizing problems: A Mendelian randomization study.	BeTwiSt project -- A representative sample of Beijing adolescents	Chinese	M; F	11–18, mean: 14.11, SD:1.83	1608	Mendelian randomization	*ALDH2* rs671	YSR	Aggression: *ALDH2* nondeficient: M = 50.37 (SD = 10.04) ALDH2 deficient: M = 49.18 (SD = 9.90), *p* = 0.033; path estimates indicated that *ALDH2* deficiency was associated with a lower alcohol use frequency, reduced quantity, and lower desire, which in turn was associated with less aggressive behavior
Zhang [54]	Monoamine oxidase A (MAOA) and catechol-O-methyltransferase (COMT) gene polymorphisms interact with maternal parenting in association with adolescent reactive aggression but not proactive aggression: Evidence of differential susceptibility.	From 39 classes in grade 6 of 14 primary schools	Jinan, China; 97.6% Chinese Han, 2.4% Chinese minorities	M; F	12–13, mean: 12.32	1399 (47.2% F)	Candidate gene, GxE	*COMT* rs4680 (Val158Met), *MAOA* rs6323 (T941G)	Proactive and Reactive Aggression Questionnaire (teacher)	Significant Val158Met x positive parenting on reactive aggression: Met carriers + positive parenting < Val/Val + positive parenting. N.S. interaction between Val158Met x parenting on proactive aggression. Significant T941G x positive parenting on reactive aggression: T alleles/TT homozygotes + positive parenting < G alleles/GG homozygotes + positive parenting. N.S. interaction between T941G x parenting on proactive aggression.
Bryushkova [103]	FKBP5 interacts with maltreatment in children with extreme, pervasive, and persistent aggression.	Children with clinically high aggression and age and sex matched controls; CAMP and Generation R Dutch birth cohort	European	M; F	6–16	170 HA and 170 HC, S2: 373	Candidate gene, case-control	*FKBP5* (5 SNPs)	CBCL	NS: case-control, CBCL aggression GxE: rs4713916 A-carriers with maltreatment had highest scores
Trucco [163]	Susceptibility effects of GABA receptor subunit alpha-2 (GABRA2) variants and parental monitoring on externalizing behavior trajectories: Risk and protection conveyed by the minor allele.	Michigan Longitudinal Study: adolescents with different risk of alcohol use disorder	96.8% European	M; F	Longitudinal, every 3 years: 3–5; Wave 2: 6–8; Wave 3: 9–11, up through 15–17	504 (359 M, 145 F)	Candidate gene, GxE, prospective study	*GABRA2* (3 SNPs)	YSR (12-17 yrs)	GxE: *GABRA2* x Parental Monitoring: GG genotype demonstrate hightened susceptibility to parental monitoring; A carriers unaffected Significant simple slope of parental monitoring for GG genotype (2.12, *p* < 0.05), NS for A-carriers (0.37, *p* = 0.22)
Musci [164]	Evaluating the genetic susceptibility to peer reported bullying behaviors.	Children from a longitudinal control trial of a bullying preventive intervention in Baltimore, conducted by the Johns Hopkins Prevention Intervention Research Center	84.9% African American, 13% White, 1% Asian and Hispanic American	M; F	mean: 6.2, SD: 0.37	561 (54.4% M)	GWAS, PRS	N/A	Peer Assessment Inventory (PAI, Pekarik et al., 1976)	PRS related to class membership, with individuals in moderate bully-victim profile group having highest level of PRS and those in high bully-victim profile having lowest levels.
Mick [109]	Genome-wide association study of the child behavior checklist dysregulation profile.	Massachusetts General Hospital, families with ADHD children	US	M; F	6–17, mean: 10.8, SD: 3.2	341 (UCLA 128, MGN 213)	GWAS	N/A	CBCL	No genome-wide statistically significant associations but identified several plausible candidate genes at *p* < 5E–05: *TMEM132D, LRRC7, SEMA3A, ALK*, and *STIP1*.
Hirata [165]	Possible association between the prolactin receptor gene and callous-unemotional traits among aggressive children.	Children with clinically high aggression - CAMP sample	77.6% Caucasian, 5.6% African-Canadians, 16.7% mixed ethnicity	M; F	6–16, mean:10.6, SD:2.92 years	96 M and 27 F cases and their matched controls	Candidate gene, case-control	*PRL*/*PRLR* (5 SNPs)	CBCL, TRF, PSD	NS for *PRL, PRLR* SNPs, or two-marker haplotypes
Galán [55]	The interaction between monoamine oxidase A and punitive discipline in the development of antisocial behavior: Mediation by maladaptive social information processing.	Pitt Mother and Child Project: longitudinal study of child vulnerability and resilience in low-income, high-risk youth	53% European, 36% African American, 5% biracial, 6% others (Asian or Hispanic)	M	Longitudunal: 1.5, 10, 17, 20, 22	187	Candidate gene, longitudinal prospective study	*MAOA* VNTR (low activity = 2, 3, 5 R; high activity = 3.5, 4 R)	Age 17: Attitudes Towards Violence Scale Age 10: official arrests, self-reported engagement in antisocial behavior; hostile attributional bias and aggressive response generation	GxE: African American and Caucasian: 2,3,5 R + increased maternal punitive discipline at age 1.5 -> inrceased aggression at age 10 (*p* < 0.001)
Zhang [51]	Gene-gene-environment interactions of serotonin transporter, monoamine oxidase a and childhood maltreatment predict aggressive behavior in Chinese adolescents.	Local middle school students	Chinese	M	mean:15.6, SD: 1.82	546	Candidate gene, GxG	*5-HTTLPR, MAOA* VNTR (low activity = 2, 3 R; high activity = 3.5, 4 and 5 R)	YSR	Adolescent aggression: NS GxE for *MAOA / 5-HTTLPR* with childhood maltreatment experience Three-way interaction: *MAOA-5-HTTLPR*-Sexual Abuse (beta=0.127, t = 2.458, *p* = 0.014) High *MAOA* activity (3.5, 4, 5 R) -> significant SA x 5-*HTTLPR* (beta=0.327, t = 3.483, *p* = 0.001) Male with high *MAOA* activity, 5-*HTTLPR*-SS and increased SA -> highest aggression
Gillentine [166]	CHRNA7 deletions are enriched in Risperidone-treated children and adolescents.	Risperidone-treated children and adolescents	USA	M; F	mean: 12.3, SD: 2.3	218 (90% M)	Candidate gene	*CHRNA7* CNVs	Clinical data including CBCL	*CHRNA7* dedeletion probands had significantly higher T scores than copy-neutral probands, even after accounting for age, sex, and weight-adjusted dose of risperidone and psychostimulants (b = 13.7, SE = 6.4, Cohen’s d = 1.26, *p* = 0.0340 for aggression)
Kiive [167]	Stressful life events increase aggression and alcohol use in young carriers of the GABRA2 rs279826/rs279858 A-allele.	Estonian Children Personality, Behavior and Health Study	Caucasian subjects, Estonia	M; F	longitudinal (ages 9, 15, 18, and 25)	583 (9 y/o), 483 (15 y/o), 457 (18 y/o), 441 (25 y/o)	Candidate gene, longitudinal, GxE	*GABRA2* (2 SNPs)	Aggressiveness subscale of 7-point Hyperactivity Scale (teacher report)	No *GABRA2* main effect on aggressive behaviors at ages 9 and 15 NS interaction between *GABRA2* and SLE on aggression at 15
Laas [91]	Nice guys: Homozygocity for the TPH2 -703G/T (rs4570625) minor allele promotes low aggressiveness and low anxiety.	Longitudinal Estonian Children Personality, Behavior and Health Study	Estonia: all Caucasian	M; F	ages 9, 15, 18, 25	583 (age 9) 483 (age 15) 454 (age 18), 440 (age 25)	Candidate gene, GxE, longitudinal	*TPH2* rs4570625, 703 G/T	ADHD Teacher’s Report	NS genotype differences in teacher-rated aggressive behavior at any age
Wang [116]	Evidence for two genetically distinct pathways to co-occurring internalizing and externalizing problems in adolescence characterized by negative affectivity or behavioral inhibition	WIC programs	10% Latino 30% African American 48% European American, 5% Native American, 1% Asian and 6% other or unknown race	M; F	14 (internalizing and externalizing problems assessed)	515 (50% F)	PRS	N/A	CBCL, TRF	Higher aggression PRS indirectly predicted primary caregiver- and teacher-reported co-occurring problems relative to all other groups through greater early childhood negative affectivity. Lower aggression PRS and higher internalizing PRS indirectly predicted co-occurring problems relative to the externalizing only and low problem groups (primary caregivers only) through greater early childhood behavioral inhibition.
Brevik [112]	Genome-wide analyses of aggressiveness in attention-deficit hyperactivity disorder.	Child ADHD sample from International Multicentre ADHD Genetics (IMAGE) study	adult ADHD: Germany, Norway, Spain; all Caucasian; child ADHD: across Europe	M; F	Child sample: 5–17	1,060 adult patients (45.1% F), 750 children & adolescents (12.5% F) with ADHD	GWAS	N/A	adult ADHD - Wender Utah Rating Scale retrospective assessment of childhood symptoms of ADHD in adults; child ADHD - Conners Parent Rating Scale (CPRS-R:L)	NS genome-wide SNPs for either individual samples or meta-analysis. Top SNPs rs10826548 in long noncoding RNA in chr 10 (beta = –1.66, se=0.34, *p* = 1.07e–6), rs35974940 in neurotrimin (*NTM*) gene (beta=3.23, se=0.67, *p* = 1.26e–6). Top gene WD repeat domain 62 (*WDR62*; *p* = 4.84e–5).
Musci [117]	Violence exposure in an urban city: A GxE interaction with aggressive and impulsive behaviors.	John Hopkins Prevention Intervention Research Center -- interventions for aggressive / shy behaviors and academic achievement	Baltimore, 85.2% were African American	M; F	longitudinal - baseline is grade 1; grades 9, 10, 11, 12	404 (55.6% M)	GxE, GWAS, PRS, longitudinal	N/A	The Teacher Observation of Classroom Adaptation-Revised (TOCA-R); Teacher Report of Classroom Behavior Checklist (TRCBC)	Significant GxE: Those with below average polygenic score and were exposed to violence more likely to be in the moderately high aggressive and impulsive class as compared to the no to low aggressive and impulsive class.
Liu [168]	Association of Y-linked variants with impulsivity and aggression in boys with attention-deficit/hyperactivity disorder of Chinese Han descent.	Han Chinese boys with ADHD	Chinese Han	M	Cases: 6–16 years (mean:9.8, SD: 2.5) years. Control: mean:16.3, SD: 8.8	857 M with ADHD and 574 male controls	Candidate gene	14 Y-linked biallelic markers, *MAOA* rs1137070	CBCL	Y-linked variants M175/DEL, M119/G showed higher ‘Inhibit’ score and aggression. Boys with *MAOA* rs1137070 T allele and the risk alleles of Y-linked markers (M88/G, M95/T, M175/DEL, and M119/G) showed the severest deficits in inhibition function and highest aggression behaviors.
Wang [64]	Interacting effect of catechol-O-methyltransferase (COMT) and monoamine oxidase a (MAOA) gene polymorphisms, and stressful life events on aggressive behavior in Chinese male adolescents.	High school students in Shandong Province, China	97% Chinese Han	M	mean: 15.26, SD: 0.29	658	Candidate gene, GxG, GxE	*MAOA* rs6323 (T941G), *COMT* rs6267 (Ala22/72Ser)	CBCL, TRF	NS main effect of *MAOA* & *COMT* on male aggression Two-way interaction: interpersonal problems x *MAOA* on teacher-reported aggression (*p* = 0.01): low activity *MAOA* (T allele) + increased interpersonal problems -> increased aggression Three-way interaction: *COMT* x *MAOA* x academic pressure (p = 0.02): lower activity *COMT* (GT/TT) + *MAOA* T allele + increased academic pressure -> increased aggression
DiLalla [169]	Gene-environment correlations affecting children’s early rule-breaking and aggressive play behaviors.	Southern Illinois Twins/Triplets and Siblings Study. Twin pairs	US; 95% Caucasian, 1% African American, 4% other	M; F	5 years (follow-up between ages 6 and 16 years)	118 twin pairs, 97 in follow-up	Twin, GxE, longitudinal	N/A	Play behavior observation and CBCL	Modest association between genetic factors related to rule-breaking behaviors and aggression (but not assertiveness) exhibited by an unfamiliar peer during free play. Genetic risk index for rule-breaking significantly correlated with aggressive behaviors in unrelated peers at baseline (*p* = 0.004). The stability of rule-breaking behaviors was largely explained by environmental factors, NS interaction with zygosity (t(47) = 1.70, *p* = 0.096).
Shao [107]	Effect of the interaction between oxytocin receptor gene polymorphism (rs53576) and stressful life events on aggression in Chinese Han adolescents.	Genetics of Mental Health study, 2nd wave subset	Han Chinese	M; F	14–17, mean:15.60, SD:1.28	197 (54 M, 143 F)	Candidate gene, GxE	*OXTR* rs53576	12-item version of the Buss and Perry Aggression Questionnaire (AQ, self-reported)	Significant interaction between *OXTR* rs53576 and life stress (F = 2.449, *p* = 0.043, partial η2 = 0.051) and of sex × SNP × life stress (F = 3.144, *p* = 0.016, partialη2 = 0.064). High life stress during the past 12 months was associated with high levels of physical aggression and hostility in *OXTR* rs53576 homozygous AA adolescents but not in G-carrier adolescents. In boys, homozygous AA individuals in the high life stress group had significantly higher levels of physical aggression. NS for girls.
Cecil [127]	Neonatal DNA methylation and early-onset conduct problems: A genome-wide, prospective study.	Avon Longitudinal Study of Parents and Children (ALSPAC)	UK	M; F	4–13 (4,7,8,10,12,13)	321 (50% F)	Candidate genes, methylation, longitudunal, EWAS	*MAOA* [14 probes], *COMT* [22 probes], *SLC6A3* [52 probes],*DRD2* [22 probes], *DRD4* [21 probes], *SLC6A4* [14 probes], *HTR1A* [14 probes], *HTR2A* [25 probes], *TPH1* [4 probes], *TPH2* [18 probes]; *NR3C1* [35 probes], FKBP5 [32 probes], *AVP* [14 probes], *OXTR* [17 probes], *BDNF* [73 probes]	SDQ ‘conduct problems’ subscale	Seven differentially methylated sites (q-value < 0.05) between early-onset vs low CP groups Three of the sites had genetic annotations: (i) cg02503763in the promoter regulatory region of *MGLL*, a gene implicated in endocannabinoid signaling and nociperception ; (ii) cg21579239, annotated to the 5’UTR region of *TTBK2*, a gene involved in tau protein phosphorylation, of which mutations have been linked to neurodegenerative disorders; and (iii) cg16006965 in the promoter regulatory region of *GCET2*, a gene implicated in immune function. The other four differentially methylated sites distal to annotated transcripts: cg12628061 located between *AK127270* (-407kb) and *PPAP2B* (-507kb), cg02131674 located closest to *BASP1* (-123kb), cg15384400 located between *ARAP2* (-12kb) and *DTHD1* ( + 26 kb), and cg07128473 located between *TRPS1* (-210kb) and *EIF3H* ( + 876 kb). In all cases, except for probe cg15384400, methylation of these sites was lower in the early-onset vs low CP groups. Candidate: *BDNF* (cg01225698, cg18354203), *FKBP5* (cg07061368) and the *MAOA* promoter region (i.e. TSS200; cg05443523)
Glenn [108]	Oxytocin receptor gene variant interacts with intervention delivery format in predicting intervention outcomes for youth with conduct problems.	Children from an RCT study comparing group and individual formats of Coping Power	197 African American; excluded 60 Caucasian/Hispanic/Others	M; F	9–11 (Mean = 9.75) at initial assessment	360 in RCT (257 provided DNA; M = 126, F = 71)	Candidate gene, longitudinal	*OXTR* rs2268493	Externalizing Composite scores from the Behavior Assessment System for Children (BASC)	A/A genotype demonstrated reductions in externalizing problems regardless of intervention format. G-allele carriers in group-based intervention showed little improvement during the intervention and a worsening of symptoms during the follow-up year, while those receiving the individual format demonstrated reductions in externalizing problems.
Paes [96]	Association between serotonin 2 C receptor gene (HTR2C) polymorphisms and psychopathological symptoms in children and adolescents.	Outpatient psychiatric unit of the Universidade Estadual de Campinas (UNICAMP) Hospital	Brazilian sample (otherwise unspecified) -- from Amilton dos Santos Júnior et al 2015 (https://www.liebertpub.com/doi/10.1089/cap.2015.0094): >50% white, then others -- mixed, black, yellow skin colors	M; F	8–18	85 (65 M)	Candidate gene	*HTR2C* rs6318	CBCL	The presence of the G allele was found to be associated with aggressive behavior (*p* = 0.033), and aggressive behavior was found to be associated with heterozygosis in females.
Womack [170]	Genetic moderation of the association between early family instability and trajectories of aggressive behaviors from middle childhood to adolescence.	Early Steps Multisite Study	27.9% African American, 50.1% European American, 13.0% biracial, and 8.9% other races (e.g., American Indian). 13 percent of participants identified as Hispanic.	M; F	2–5 years (initial); trajectories of aggression identified from 7.5–14 years.	515 (49% F)	PRS, longitudunal	N/A	CBCL, TRF	The family instability by polygenic interaction predict growth in child aggression: children with lower levels of family instability and lower polygenic risk had steeper decline in aggression from 7.5 to 14. Simple slopes indicate that children experiencing lower instability (1 SD below the mean) demonstrated a shallower initial decline in aggression at higher aggression PRS (B = 0.35, SE = 0.216, p = 0.028). Significant positive effect of the interaction term on the quadratic growth of aggression. Simple slopes revealed that at low instability (1 SD below the mean), children with higher aggression PRSs demonstrated steeper negative quadratic growth (B= −0.05, SE = 0.02, *p* = 0.011).
Pappa [35]	A genome-wide approach to children’s aggressive behavior: The EAGLE consortium.	EAGLE consortium, 9 population-based studies	North European ancestry	M; F	early childhood 3–7 years; middle childhood / early adolescence 8–15 years	18,988 (N = 15,668 early childhood and N = 16,311 middle childhood/early adolescence)	GWAS	N/A	SDQ, CBCL, general parent-rated quesstionaires	GCTA quantified variance tagged by common SNPs (10–54%). The meta-analysis of the total sample identified one region in chromosome 2 (2p12) at near genome-wide significance (top SNP rs11126630, *P* = 5.30e–8). The separate meta-analyses of the two developmental stages revealed suggestive evidence of association at the same locus. Gene-based analysis indicated association of variation within *AVPR1A* with aggressive behavior (*p* = 1.61e–3).
Wang [97]	Serotonin functioning and adolescents’ alcohol use: A genetically informed study examining mechanisms of risk.	The Adult and Family Development Project (AFDP) and Child Development Project (CDP)	non-Hispanic Caucasian	M; F	AFDP: 10–17.99 at T1, 11–18.99 at T2, and 13–20.99 at T3 CDP: 7th- (T1; 12–13 years old), 9th- (T2; 14–15 years), and 10th-grade (T3; 15–16 years)	AFDP 254; CDP 348	Candidate gene, PRS, longitudinal	PRS targeting serotonergic genes	CBCL-YSR	5-*HT* polygenic risk did not predict self-regulation but adolescents with higher levels of 5-*HT* polygenic risk showed greater depression and aggression/antisociality. Adolescents’ aggression/antisociality mediated the relation between 5-*HT* polygenic risk and later alcohol use. Deficits in self- regulation also predicted depression and aggression/antisociality, and indirectly predicted alcohol use through aggression/antisociality.
Vestlund [171]	Ghrelin and aggressive behaviors-Evidence from preclinical and human genetic studies.	CATSS, an on-going nation-widestudy targeting all twins born in Sweden since July 1992	Caucasian	M	18	784	Candidate genes, twin	*GHRL* (2 missense SNPs), *GHSR* rs2948694	Self-Reported Delinquency questionnaire (SRD)	No SNPs associated with overt aggression. Significant interaction for *GHRL* rs696217 with risk-drinking on overt aggression (F (1717) = 7.99, *p* = 0.00482). Met allele (Leu72Met + Met72Met) and having hazardous alcohol use displayed lower levels of aggression than risk-drinkers who did not have the Met allele (Leu72Leu, *p* = 0.011)
Van Hulle [42]	Sex differences in the genetic and environmental influences on self-reported non-aggressive and aggressive conduct disorder symptoms in early and middle adolescence.	The Tennessee Twin Study (TTS) sample	71.4% Non-Hispanic European-American, 24% African-American, 4.6% mixed / other ethnic	M; F	9–17: grouped into ages 9–13 years and 14–17 (M = 12.8, SD = 2.6)	1,548 pairs of twins	Twin	N/A	Child and Adolescent Psychopathology Scale, Self-report CD	The same genetic and environmental factors influenced CD symptoms in both sexes, but the total variability was lower in females than males. Early to middle adolescence: non-shared environment accounted for the majority of the variance for both non-aggressive (58%) and aggressive (69%) CD symptoms. Significant shared environmental influences on non-aggressive CD symptoms (31%) and marginal genetic influences (11%). Marginal shared environmental influences (11%) and modest genetic influences (20%) on aggressive CD symptoms. Middle adolescence: genetic influences on non-aggressive CD were robust (39%) with modest shared environmental influences (13%). Genetic (47%) and non-shared environmental (53%) influences accounted for all of the variance in aggressive CD, with no evidence for shared environmental influences. Genetic influences accounted for less variation in early adolescence compared to middle adolescence (18% vs 47%)
Sener [95]	Altered global mRNA expressions of pain and aggression related genes in the blood of children with autism spectrum disorders.	Autism Spectrum Disorders	Turkey	M; F	3.64 (1.17) ASD and 3.64 (1.19) control	40 (ASD) and 50 age- and sex-matched controls	Candidate genes, RNA expression	*HTR1E, OPRL1, OPRM1,TACR1,PRKG1,SCN9A, DRD4, CRHR1, SLC6A4* (mRNA expressions)	N/A	*OPRM1, PRKG1*, and *HTR1E* genes low expressing and *SCN9A, DRD4, OPRL1*, and *TACR1* high expressing in ASD compared to controls. *HTR1E* gene expression between aggression positive (0.74; 0.4-1.75) and negative (2.37; 0.56-3.22) groups significant (*p* = 0.037).
Jamnik [36]	A multimethodological study of preschoolers’ preferences for aggressive television and video games.	Twin/triplet sample: Southern Illinois Twins/ Triplets and Siblings Study	reported to be Caucasian (95%), with 2.5% African American and 2.5% other.	M; F	5 year old	184 (92 families, 107 F)	Twin/triplets, heritability	N/A	Child-reported media preferences, CBCL, observed child negative behaviors (incl. aggression)	Children rated as more aggressive by their parents were more likely to have more aggressive mothers (R = 0.3, *p* < 0.001). N.S. correlations between MZ and same sex DZ indicating no evidence for heritability of preferences for physically or relationally aggressive media or on children’s negative behaviors during the parent-child interactions. Correlation between opposite sex DZ pairs for preferences for relationally aggressive media was negative and significantly less than for same sex DZ pairs (z = 2.47, *p* = 0.007) For CBCL aggression, the MZ correlations were significantly greater than the DZ correlations (z = 2.14, *p* = 0.016), with a heritability estimate of 0.60.
Slawinski [39]	The etiology of social aggression: a nuclear twin family study.	Twin Study of Behavioral and Emotional Development in Children (TBED-C)	81.7% non-Hispanic White, 9.5% African American, 1.1% Native American, 0.8% Asian, 0.7% Hispanic 0.3% Pacific Islander and 5.9% multiracial or other.	M; F	6–11, mean: 8.02, SD: 1.49	1,030 pairs (48.7% F)	Nuclear Twin Family (NTF) model	N/A	Subtypes of Antisocial Behavior Questionnaire - maternal, paternal, teacher	Social aggression: generally largely additive genetic (A = 0.15–0.77) and sibling environmental (S = 0.42–0.72) in origin Etiology of social aggression not substantially influenced by assortative mating (μ = 0.07–0.09) or the covariance between additive genetic and familial environmental effects (e.g., passive rGE = −0.14 to 0.06).
Achterberg [37]	Heritability of aggression following social evaluation in middle childhood: An fMRI study.	Longitudinal twin study of the Leiden Consortium on Individual Development (L-CID)	91% Caucasian, Dutch	M; F	7.95 ± 0.67 (age range: 7.02–9.68); MRI participants (7.99 ± 0.68)	509 (MRI sample 385 participants)	Twin studies, heritability	N/A	Social Network Aggression Task (SNAT)	Aggression following negative relative to positive social feedback was moderately influenced by genetics (A = 20%) and to a lesser extent influenced by shared environment (C = 6%). Unique environment and measurement error explained the largest part of the variance in aggression after negative feedback (E = 74%). Similar finding following negative relative to neutral feedback (A = 10% vs C = 8%), Decreased SMAand DLPFC activation during negative feedback was associated with more aggressive behaviorafter negative feedback. Moreover, genetic modeling showed that 13%–14% of the variance indorsolateral PFC activity was explained by genetics.
Liu [102]	Association of corticotropin-releasing hormone receptor-1 gene polymorphisms and personality traits with violent aggression in male adolescents.	Chinese	Chinese	M	violent crime group: 17.06 ± 0.85; non-violent crime group: 17.43 ± 0.57; normal control group: 31.17 ± 7.48	138 violent young male criminals, 98 nonviolent young male criminals, and 153 noncriminal adults	Candidate gene, case-control	*CRHR1* (2SNPs)	Modified Overt Aggression Scale	rs242924 allele frequencies differ across the 3 groups: G allele frequency higher in violent crime group vs normal adult group (OR = 2.29, 95% CI [1.13–4.62]); GG haplotype significantly higher than in normal control group vs violent crime group (*p* < 0.05, OR = 1.072, 95% CI [0.547–2.101]); rs17689966 A allele (p = 0.012, OR value 2.327, 95% CI [1.19–4.56]) and AA/AG genotype frequencies higher in conduct disorder subgroup vs non-CD subgroup within violent crime group (*p* = 0.024)
Letourneau [89]	Parenting interacts with plasticity genes in predicting behavioral outcomes in preschoolers.	Women and their infants enrolled during pregnancy in the ongoing Alberta Pregnancy Outcomes and Nutrition (APrON) cohort study. Fetal Programming Study	Maternal ethnicity 84% Caucasian, Canada	M; F	24 mos	176 with 24-month behavioral data (minus 19 with missing genotypes = 157)	Candidate genes, GXE	*DRD2* rs1800497, *BDNF* rs6265, *CNR1* (2SNPs), *DRD4* exon 3 VNTR 7 R, *DAT1* VNTR, 5-*HTTLPR*, *MAOA* VNTR (low-activity = 2, 3, 5 R)	CBCL - 24 months of age	Children with *CNR1*-A plasticity allele (p = 0.010) or *DAT1* 9-repeat plasticity allele (*p* = 0.036) and experienced more/less parental control displayed more/fewer externalizing problems, respectively, in a differentially susceptible manner.
Vaht [172]	Variation rs6971 in the translocator protein gene (TSPO) is associated with aggressiveness and impulsivity but not with anxiety in a population-representative sample of young adults.	Longitudinal Estonian Children Personality, Behavior and Health Study sample	European	M; F	Retrospectively recording about secondary school at age 33.5 SD: 0.7	655	Candidate gene	*TSPO* rs6971	Illinois Bully Scale -self-report	rs6971 AA -> highest aggressiveness, reported bulluing other students the most (F(2, 500) = 3.9, *p* = .022)
Ip [118]	Genetic association study of childhood aggression across raters, instruments, and age.	29 cohorts	European ancestry	M; F	1.5–18	87485 ( ~ 50% M)	GWAS, genome-wide association meta-analysis, PRS	N/A	Achenbach System of Empirically Based Assessment (ASEBA; 41%; Achenbach et al. 2017) and the Strengths and Difficulties Questionnaire (SDQ; 34%; Goodman 2001) most commonly used. Overall, 26 different assessments were used (see Supplementary Table 3).	No genome wide significant SNPs. Three gene-based analysis sig genes: *ST3GAL3* (*p* = 16E-06), *PCDH7* (*P* = 2.0E–06), *IPO13* (*P* = 2.5E–06). Aggression strongly genetically correlated with smoking phenotypes (rg = 0.46–0.60) In children, 11 of 16 PGSs (for GWAMA p-value thresholds) correlated to mother-reported aggression among 7-year olds.
Orri [40]	Contribution of genes and environment to the longitudinal association between childhood impulsive-aggression and suicidality in adolescence.	Quebec Newborn Twin Study	Canada, Majority Caucasian, 21% African, 3% African	M; F	6–12 (6, 7, 9, 10, and 12)	862 twins (335 MZ, 527 DZ) from 435 families	Longitudunal, twin study	N/A	Teacher rating of child behavior over the past 6 months using 4 items from Dodge & Cole (1987): overreacts angrily to accidents; blames others in fight; when teased, strikes back; reacts in an aggressive manner when contradicted.	Additive genetic factors accounted for individual differences in impulsive - aggression intercept (A = 90%, E = 10%), and slope (A = 65%, E = 35%). Genetic (50%) and unique environmental (50%) factors equally contributed to suicidality. Thirty eight percent of the genetic factors accounting for suicidality shared with those underlying impulsive-aggression slope, whereas 40% of the environmental factors accounting for suicidality shared with those associated with impulsive-aggression intercept. The genetic correlation between impulsive-aggression slope and suicidality was 0.60, *p* = 0.027.
Vollebregt [101]	Evidence for association of vasopressin receptor 1 A promoter region repeat with childhood onset aggression.	High aggression cases and non-aggressive controls -- CAMP sample	European (Canada)	M; F	Main analysis: 12.2 (2.9) for cases and 11.0 (2.6) for Controls	299 HA (185 M) and 192 NC (96 M) - RS1 201 HA and 189 NC for RS3 for main analysis	Candidate gene, case-control	*AVPR1A* RS1 and RS3	CBCL, TRF, YSR, 2+ years of aggression according to parent	Nominally significant association between one specific RS3 repeat and non-aggressive status. No association was found for any of the RS1 repeats. In a separate model, grouping repeats into short and long, carriers of long RS3 repeats were nominally significantly associated with non-aggressive status.
Nedic Erjavec [92]	Serotonin 5-HT(2 A) receptor polymorphisms are associated with irritability and aggression in conduct disorder.	Croatian youth with CD vs drug-naive controls	Croatian	M	control: 16 (15–18), CD: 17 (16–18)	185 juvenile correctional facility detainee (120 with and 65 without DSM4 Conduct Disorder) & 43 healthy adolescent controls	Candidate gene, case-control	*HTR2A* (4 SNPs)	Hare Psychopathy Checklist: Youth Version (PCL-YV), OAS-modified, CBCL	Physical aggression toward others associated with G allele of rs2070040 and rs4142900 and the C allele of rs9534511 and rs9534512.
Kant [67]	COMT Val/Met and psychopathic traits in children and adolescents: A systematic review and new evidence of a developmental trajectory toward psychopathy.	Children with clinically high aggression - CAMP sample	European	M; F	6–17: mean: 12.41, SD: 2.86	293 (142 M, 151 F)	Candidate gene	*COMT* rs4680 (Val158Met)	CBCL	Males >=13: Val-allele increased aggression (*p* = 0.03) Males <13: NS
Kant [47]	Association of the MAOA-uVNTR polymorphism with psychopathic traits may change from childhood to adolescence.	Children with clinically high aggression - CAMP sample	European	M; F	6–17: mean: 11.7, SD: 2.72	336 (194 M, 142 F)	Candidate gene	*MAOA* VNTR	CBCL	NS

The summary shows for each study the title, population characteristics, ancestry or the country in which the study was carried out, sex, average age, sample size, study type, gene assessed, assessment tool for aggression, and key findings.

## Results

Eighty-seven studies were included in our data extraction and quality assessment. The general summary of these articles can be found in Table 1. Seventy percent (*n* = 61) of the articles examined samples from European ancestry. Eighty percent (*n* = 70) of studies included both females and males, while 19% (*n* = 16) studies only included males. Only one study included only females in their study.

The first research study retrieved using our search strategy was by Twitchell and colleagues in 2001 [34], which examined the genetics of child aggression among offspring of alcoholic fathers. Until 2006, childhood aggression was examined in association with particular psychiatric disorders, such as ADHD, CD and ODD, and studies examining aggression as a transdiagnostic behavioral phenotype were rare. The first studies used the candidate gene approach, which continues to be the major research method (74% of all studies) used in studying the genetics of childhood aggression. Until 2007, studies focused on several key genes, including serotonin transporter, *MAOA*, and dopamine D4 receptor. From 2011, genome-wide association studies started to be published, although the sample size remained relatively small (n < 1000) until 2016 [35].

### Twin / pedigree studies

Throughout the years, researchers focused on twin studies for heritability and understanding the contribution of genes to aggressive behaviors. Studies have demonstrated a significant heritability for aggressive behaviors of up to 60% [36–38]. However the influence of genes can be augmented by the environment such that it decreases with decreasing positive social feedback [37] or increasing parental negativity [38]. From the genetic influences, mainly additive genetic factors have been found to explain the variability; they accounted for 15-77% of the variance in social aggression [39] and up to 90% of the individual differences in baseline impulsive aggression in the longitudinal Quebec Newborn Twin Study [40]. Interestingly, the effects of genetic factors on aggressive behaviors can change over time [41] and the influence of genes on the variation of aggressive behaviors can change over development (18% genetic influence in early-middle adolescent to 47% genetic influence in middle adolescent) [42]. Although the magnitude of the influence may change, genetic factors still account for the stability of physical aggression as well as can explain the individual differences in the initial levels and the rate of change of aggressive behaviors over time in a longitudinal Canadian sample [41].

### Candidate gene studies

The majority (77%; *n* = 67) of genetic studies in childhood aggression used a candidate gene approach. The most commonly investigated genes include monoamine oxidase A (*MAOA;* *n* = 17), dopamine D4 receptor (*DRD4*, *n* = 13), and catechol-o-methyltransferase (*COMT*, *n* = 12). Many of these candidate gene studies, however, suffer from methodological issues including small sample size (often <500), lack of psychometrically sound assessments of aggression, and inconsistencies in the cutoffs and categorization of repeat polymorphisms, thus making generalization of findings difficult.

#### Monoamine oxidase A—MAOA

The *MAOA* gene is located on the X-chromosome (Xp11.23) and encodes for MAOA, an enzyme which catabolizes monoamine neurotransmitters such as serotonin, epinephrine, norepinephrine and dopamine. In our systematic review, *MAOA* was assessed in over a quarter of the studies assessing candidate genes (*n* = 17*;* one study was categorized as epigenetic studies). The most commonly examined polymorphism is *MAOA*-uVNTR, the 30-bp repeat polymorphism, which can exist in 2 repeats (2 R), 3 R, 3.5 R, 4 R, and 5 R. Many studies regard the 2 R, 3 R, and 5 R to be low activity variants (MAOA-L), and the 3.5 R and 4 R to be high activity variants (MAOA-H) [27]. Nevertheless, there is great variation across studies in the categorization of low and high activity repeats (see Table 1), making direct comparisons of findings across studies challenging.

The first study that investigated the association between *MAOA*-uVNTR and childhood aggressive behavior in the context of ADHD reported that low transcription (3 R only) alleles of *MAOA*-uVNTR was associated with higher aggression among children with ADHD [43]. Similarly, different studies have also found that the low-expression (2 R, 3 R, and 5 R) *MAOA*-uVNTR to be associated with childhood aggression, although their categorization of low-expression alleles differ [44]. On the other hand although fewer in number, some studies have reported that the high-expression variant was associated with increased aggressive behavior in children [45].

While the reason for these inconsistent findings across studies has not been fully examined, several researchers have suggested that developmental age may have an influence on the observed effects of *MAOA* risk alleles. Pingault et al. [46] examined the age-dependent contribution of six *MAOA* SNPs on childhood physical aggression using a longitudinal dataset of 436 boys followed annually from ages 6 to 12 in Quebec, Canada.The results showed that the T-allele carriers for rs5906957 had lower initial levels of physical aggression and also a less steeper decline in physical aggression over time compared to the C allele carriers. In a similar vein, Kant et al. [47] examined the effect of *MAOA*-uVNTR on aggression and psychopathic traits by developmental age. The 194 male participants were divided into those below age 13 (*n* = 132) and those at or above age 13 (*n* = 62). While *MAOA*-uVNTR was not significantly related to aggression in either age group, there was an interesting pattern in that, in the younger age group, oppositional defiant problems and conduct problems were associated with the high-activity *MAOA* 4 R allele (MAOA-H), whereas in the older age group, oppositional defiant problems and callous-unemotional traits were more significantly associated with the low-activity *MAOA* 3 R allele (MAOA-L). More studies are required to confirm the age-dependent association of MAOA on aggressive behavior.

It should be noted, however, that the majority of studies reported no significant main effects for *MAOA* gene variants on childhood aggression [48]. Despite this, following the example of Caspi et al.’s [28] seminal study which reported no significant main effect of *MAOA*-uVNTR but a significant *MAOA* gene by childhood maltreatment interaction, several studies examining gene-by-environment interaction have been conducted. Various types of environmental exposure have been examined, including child maltreatment, abuse [49–51], and parenting behaviors [52–55]. These gene-environment interaction studies have sometimes been used to test theories regarding the role of genes and environment in childhood aggression. For example, Zhang et al. [54] sought to test two related hypotheses regarding the role of gene and environment: Diathesis-stress (i.e., carriers of certain genetic risk variants will show greater aggression when exposed to adverse environments) and differential susceptibility (i.e., not only do carriers of certain genetic variants show greater aggression when exposed to adverse environments, the carriers of the same genetic variants will show less aggression when exposed to supportive environments). This study with 1399 healthy Han Chinese adolescents supported the differential susceptibility hypothesis; males who had the T allele and females who had the homozygous for the T/T genotypes for *MAOA* rs6323 (T941G) were more likely to exhibit reactive aggression when the mothers exhibited low levels of positive parenting but were less likely to exhibit reactive aggression when mothers exhibited high levels of positive parenting.

#### Catechol-O-Methyltransferase—COMT

Catechol-o-methyltransferase (COMT) is an enzyme that metabolizes catecholamine neurotransmitters including dopamine, epinephrine, and norepinephrine. It has two isoforms, a longer membrane-bound (MB-COMT) isoform that is expressed mainly in neurons in the brain [56], and a shorter soluble (S-COMT) isoform that is expressed in other tissues such as blood, liver, and kidney. It is coded by the *COMT* gene, which is localized on chromosome 22q11.21. A common single-nucleotide polymorphism, rs4680 (Val158Met), within the coding region of *COMT* changes the amino acid Valine at position 158 of MB-COMT to Methionine, which decreases the thermostability and activity of COMT enzyme. The role of COMT in aggression was initially supported by observations of hostility in mice deficient in *Comt* and the negative correlation between COMT levels and hostility in men with behavioral problems as children [57, 58]; reviewed in Qayyum et al. (2015) [59].

The *COMT* genetic variants, particularly rs4680, have received a similar level of attention as *MAOA* in association studies of child aggression. The first published study that examined a possible association between *COMT* gene and child aggression was in the context of ADHD, where Caspi et al. [60] reported an association among ADHD patients of Val/Val with increased aggression compared to Met-carriers; the association was replicated across three samples within this study. Other studies reported high aggression being associated with either Met-allele carriers [61], or no significant association [62]. Few studies examined SNPs other than rs4680, with one reporting rs6269 A/G heterozygotes being over-represented in cases compared to adult controls [63] and another reported non-significant results for rs6267 (Ala22/72Ser in S/MB-COMT) [64].

The possible association of *COMT* with child aggression has been examined in the context of interactions with environmental or demographic variables. For example, in a birth cohort study, among children who scored high on disorganized attachment, Val/Val carriers exhibited greater increase in aggression from 4 years to 6 years of age than Met-allele carriers [65]. The same research group also reported that, among those with stressful life events, rs4680 Val/Val homozygotes were more aggressive than Met-allele carriers, while the reverse was observed among those without stressful life events [66]. In a study on Chinese early adolescents, Val/Val carriers were reported to display higher reactive aggression compared to Met-allele carriers in the context of higher positive parenting scores, but lower reactive aggression compared to Met-allele carriers with lower positive parenting scores [54]. Age and sex may also be an effect modifier for the effect of rs4680 on risk of child aggression. For example, Kant and colleagues [67] demonstrated that among European males at least 13 years of age, Val-allele carriers had higher CBCL aggressive scores than non-carriers (*p* = 0.03). In contrast, among those younger than 13, Met/Met genotype carriers had increased conduct problems compared to Val-allele carriers (*p* = 0.03). These associations were not observed in females in their sample. A three-way interaction was reported, where carriers of the *COMT* low-activity rs6267 T allele and *MAOA* rs6323 T allele displayed higher aggressive behavior in the presence of high academic pressure than those with low academic pressure; this association was not observed in carriers of other genotype combinations [64]. Further efforts in large samples are needed to confirm these preliminary interaction findings and pursue more complex interaction analyses.

#### Dopamine system genes

The dopamine system is vital to the regulation of motor and cognitive behaviors, and dopamine dysregulation has been implicated in multiple psychiatric and behavioral disorders.

Within the dopamine system, aside from *COMT* mentioned above, the most studied dopamine system gene in child aggression is the dopamine D4 receptor-encoding *DRD4*, which is localized on 11p15.5. The 48-bp exon III variable number tandem repeat (VNTR) polymorphism [68, 69] is the extensively studied *DRD4* polymorphism, for which between two to eleven repeats (R) have been observed in humans, with the 4-repeat (4 R), 2 R, and 7 R being the most commonly observed alleles. Functional significance of this polymorphism has been demonstrated [70–75]. The 7 R has been shown to reduce in-vitro DRD4 expression [73] and to be less likely to form heterodimers with the dopamine D2 receptor [76], while the 4 R allele appears to be less responsive to quinpirole-mediated DRD4 upregulation [74].

The larger repeat (7 R or 6-8 R) alleles were associated with high aggression in an Italian sample [77], the Mannheim Study of Children at Risk study [78], and the Ben-Gurion University Infant Developmental Study [79], while the 3 R allele (*p* = 0.014) and rs3758653 C/C genotype were nominally associated with aggressive behavioral impulsivity in the International Multicenter ADHD Genetics (IMAGE) study [80]. The VNTR was not associated with externalizing behavior in a longitudinal community sample of 87 boys [81], within our earlier sample of 48 clinically referred aggressive boys [81], or our later sample of 144 high aggression child cases and adult controls [82].

A number of gene-environment interaction findings have been reported for *DRD4*. In a study on the Dutch Twin Registry sample, a significant VNTR-by-maternal sensitivity interaction was observed. More specifically, larger repeat allele (7 R or 6-8 R)-carrying genotypes were associated with higher externalizing behaviors or aggression compared to 7 R non-carrying genotypes (e.g., 2–4 R) only in the context of maternal insensitivity [83], high maternal prenatal stress [84], or low-aggression peer play environment [85]. Besides *COMT* and *DRD4*, only few other dopamine system genes have been examined in child aggression, with our group reporting that *DRD2* rs1799978 (A-241G) G-allele carrying genotypes, rs1079598 C/C genotype, and rs1800497 (TaqIA) T/T genotype were overrepresented in high aggression cases compared to adult controls [82]. A significant *DRD4*-by-socioeconomic status interaction in high aggression scores has been reported, where the 6–8 R carriers with low socioeconomic status had higher aggression scores compared to other comparison groups [77].

#### Serotonin system genes

Under the serotonin system genes, the most extensively studied polymorphism is the 5-hydroxy-tryptamine-linked polymorphic region (5-HTTLPR) polymorphism of the *SLC6A4* gene.

5-HTTLPR long allele (L/L genotype) was associated with higher CBCL aggressive behaviors score in 607 Italian children [77]. Interaction between low socioeconomic status and 5-HTTLPR long alleles further demonstrated significant effects on aggressive behaviors [77]. A smaller sample consisting of 62 European participants similarly reported an increased risk for behavioral disinhibition and aggressive behaviors with the L/L genotype when compared to S/S and S/L [34]. On the other hand, Beitchman and colleagues [86] reported a significant effect of 5-HTTLPR on aggression with the low expressing (S/S, Lg/S, Lg/Lg) genotypes in children with clinically severe aggression. Similarly, the S-allele was significantly associated with teacher reported aggressive behaviors at age 9 for both boys and girls [87] and with increased aggressive behaviors and hostility in a group of female Caucasian Russian swimmers [88].

There are also studies that did not yield significant 5-HTTLPR main effect findings on childhood aggression. Several studies on European children and adolescents [78, 89] and Chinese adolescents [51] did not report a significant main effect of 5-HTTLPR on aggressive behaviors and related phenotypes. Similarly, the initial analyses of the study on 87 adopted children from the United States of America further failed to detect a main effect of 5-HTTLPR and aggressive scores [90]. However interestingly, when the biological parent status and sex of the children were included in the analyses, the results were significant. Male children with S/S or S/L (short) demonstrated increased aggressive behaviors while females with the SS and SL demonstrated lower levels of aggression. Moreover, when the biological parent of the child was considered antisocial, adolescents, but not preadolescents, demonstrated a significant increase in aggressive behaviors with the L/L genotype [90].

Although some studies failed to report a significant main effect of 5-HTTLPR on aggressive behaviors, they demonstrated a significant gene-gene interaction on behavior. Zhang and colleagues [51] reported that there was a three-way interaction between *MAOA* high activity, 5-HTTLPR and sexual abuse on aggressive behaviors. Children with *MAOA* high activity, 5-HTTLPR S/S allele and with increased sexual abuse experience exhibited higher aggressive behaviors [51]. Furthermore, there was a significant interaction between 5-HTTLPR S/S genotype and *DRD4* 7 R on increased aggression scores [78], while Nobile and colleagues [77] demonstrated increased aggression with *DRD4* VNTR 6-8 R and 5-HTTLPR L/L genotype.

Other polymorphisms of serotonin system genes that have been studied in relation to childhood aggression include *SLC6A4* VNTR polymorphism and *tryptophan hydroxylase 2* (*TPH2)* gene polymorphisms. Neither the *SLC6A4* VNTR [86] nor the *TPH2* rs4570625 polymorphism [91] demonstrated significant associations with childhood aggression. Furthermore, four SNPs of the *5-hydroxytryptamine receptor 2* *A (HTR2A)* gene that encodes for one of the receptors for serotonin failed to have a significant difference between the conduct disorder cases and controls in adolescents [92]. However, in adolescent cases, G/G genotype or the G allele carriers of rs2070040, C-allele carriers of rs9534511 and G-T haplotype of rs2070040- rs9534511 were associated with increased aggressive scores [92]. On the other hand, in adolescent controls T/C haplotype of rs4142900-rs9534512 was associated with the increased aggressive behaviors [92]. Other serotonin receptor encoding gene polymorphisms, including *5-hydroxytryptamine receptor 1B* [93, 94]*, 1E* [95] and *2* *C* [96] further demonstrated significant effects on childhood aggression **(**Table 1**)**. Lastly, a recent comprehensive study analyzing the association between polygenic score indexing serotonin functioning and aggression demonstrated that adolescents with higher serotonin polygenic risk (lower levels of serotonin functioning) had an increased risk for aggressive and antisocial behaviors [97].

#### Hypothalamic-pituitary-adrenal (HPA) axis and hormonal signaling genes

HPA axis refers to the neuroendocrine system that involves the hypothalamus, pituitary, and adrenal glands and is responsible for stress response and regulation of various biological processes such as food digestion and immune response. The hypothalamus secretes corticotropin-releasing hormone (CRH) and arginine vasopressin (AVP) in response to physical or psychological stress. These two neurohormones are transported to the pituitary through blood vessels and bind to the CRH and AVP receptors respectively and stimulate the release of adrenocorticotropic hormone (ACTH). ACTH then stimulates the secretion of glucocorticoids such as cortisol. Glucocorticoids in turn provide a negative feedback signal to inhibit the secretion of CRH and AVP from the hypothalamus and ACTH from the pituitary gland, respectively. Animal studies have consistently shown a robust association between the HPA axis and aggressive behavior [98]. Nevertheless, relatively few studies have interrogated genes along the HPA axis with regards to childhood aggression.

Several researchers have investigated the arginine-vasopressin related genes in association with childhood aggression. Zai et al. [99] examined eleven SNPs from the AVP receptor 1 A (*AVPR1A*), *AVPR1B*, and *AVP* genes in 177 children with high aggression and ethnicity and sex matched adult controls and found a significant association between childhood aggression and *AVPR1B* rs35369693, as well as the two-marker haplotype containing rs35369693 and rs28676508. Similarly, Malik et al.’s [100] study compared 182 clinically aggressive children of European ancestry with 182 sex, age and ancestry-matched non-aggressive controls and found that the A allele and the AA genotype of the rs3761249 SNP of the *AVP* gene was underrepresented in highly aggressive male cases, whereas *AVPR1A* rs1174811 G allele was over-represented in highly aggressive female cases. While the former studies examined SNPs in the AVP pathway, Vollebregt et al. [101] examined two microsatellites, RS1 and RS3, of the *AVPR1A* gene among children with pervasive aggression and non-aggressive age-matched controls. They found that the RS3 long repeat variants were nominally associated with non-aggressive status. It is noteworthy that Pappa et al.’s [35] genome-wide association study also found an association between the *AVPR1A* gene and childhood aggressive behavior in a post-hoc gene-based analysis, warranting further investigations of *AVPR1A* gene variants.

With regards to the corticotropin releasing hormone (CRH), Liu et al. [102] reported that the carriers of the G allele and the GG genotype for rs24924 of the corticotropin-releasing hormone receptor *CRHR1* gene were overrepresented among young offenders of violent crime compared to non-violent control adults in a Han Chinese sample.

The *FKBP5* is a co-chaperone of the glucocorticoid receptor. Studying the association of the *FKBP5* gene with childhood aggression, Bryushkova et al. [103] did not find significant main effects with any of the SNPs within the gene, but found a significant gene-environment interaction in that A allele carriers of the *FKBP5* rs4713916 who were exposed to maltreatment exhibited the highest levels of aggression.

Oxytocin (OXT) is a nonapeptide most widely known for its stress-reducing effects and has been shown to affect prosocial behaviors, emotional recognition and feelings of trust [104–106]. The gene coding for OXT receptor (*OXTR*) has been examined in association with childhood aggression. The study by Malik et al. [100] found that *OXTR* rs237898 A allele was over-represented in high aggression children. Other studies have found a gene-by-environment interaction between variants in the *OXTR* gene and stressful life events [107]. Glenn et al., [108] found that the variations in the *OXTR* gene moderated the effectiveness of, Coping Power, a disruptive behavior modification program.

### Genome-wide association studies

We have identified 12 genome-wide association studies (GWASs) of child aggression, the majority of which were performed on children and adolescents with European ancestry. The first GWAS focused on the Dysregulation Profile from CBCL (which consists of Attention Problems, aggressive behavior, and anxious/depressed clinical subscales) among 341 ADHD children from 339 ADHD affected trio families [109]. This study found no genome-wide statistically significant associations (*P* < 5 × 10^–8^); however, *TMEM132D*, *LRRC7*, and *STIP1* were identified as nominally significant [109]. The second GWAS on 398 ADHD child cases from Cardiff and 5,081 controls from the Wellcome Trust Case Control Consortium (Phase 2) found higher polygenic risk scores for ADHD (ADHD-PRS) scores in ADHD cases with diagnosis of conduct disorder compared to those without, and positive correlation between ADHD-PRS and the number of aggressive conduct disorder symptoms within cases [110]. The first GWAS by the EAGLE (Early Genetics and Lifecourse Epidemiology) Consortium performed quasi-Poisson regression on aggression scores across nine cohorts with a total of 18,988 participants from early childhood and mid-childhood/ early adolescence and reported a near genome-wide significant variant (rs11126630, *P* = 5.3e–8) at 2p12 and a significant gene (*AVPR1A*) [35]. They also reported that the 450,000 tested common variants accounted for between 10% and 54% of the variance in aggression across three sample sets ranging from 3 to 6 years of age [35]. The authors suggested that the large range of observed SNP heritability could be due to different sample characteristics, environmental contributions, and ages across these samples [35]. With additional samples across the age ranges, we may be able to capture the pattern of genetic components across development. [35] The authors followed up with GWAS of aggression subtypes as well as a cross-trait gene-based meta-analysis of GWAS of aggression with GWAS of volume of amygdala, nucleus accumbens, or caudate nucleus [111]. They found the *MECON* (MDS1 And EVI1 Complex Locus) gene to be associated with cross-trait construct of aggression and nucleus accumbens volume, and the *AVPR1A* gene to be associated with the construct of aggression and amygdala volume. Another GWAS of aggressiveness during childhood on 1050 adult ADHD patients and 750 child ADHD patients reported the top suggestive variant in a long non-coding RNA gene on chromosome 10 (rs10826548) and the top suggestive gene to be WD repeat domain 62 (*WDR62*) [112].

A Polygenic risk score (PRS) is an estimate of the genetic risk for a phenotype of interest and is generally calculated based on the number of risk alleles each person possesses and the effect sizes of these risk alleles [113]. Genetic correlation is an estimate of the genetic similarity between two complex phenotypes by calculating the correlation of phenotypic effects across genetic variants [114]. In a longitudinal study of children of diverse low-income families from the Women, Infants, and Children Nutritional Supplement Programs (WIC) study, PRS for child aggression [35] based on all SNPs with *p* < 0.05 or SNPs mapped to gene regions were not significantly associated with aggression at any age from early to mid-childhood, while PRSs enriched for SNPs with putative biological function were associated with aggression, with effect estimate appeared to change through early childhood (age 2–5 years) to mid-childhood (age 7.5–10.5 years) [115]. In a more recent study on the WIC sample, higher aggression-PRS based on the EAGLE Consortium GWAS [35] appeared to predict greater co-occurring internalizing/externalizing problems at age 14 via negative affectivity observed during parent-child play at age 3 [116]. In a sample of 404 participants from a school-based program consisting of two preventive interventions for early learning and aggressive/ disruptive behaviors, polygenic risk scores for conduct disorder from the SAGE (Study of Addiction: Genes and Environment) sample, an interaction between polygenic risk scores and exposure to community violence was observed such that among those who endorsed witnessing violence, conduct disorder PRS was negatively associated with likelihood of being in the high-aggression group (or positively associated with likelihood of being in the lowest aggression group [117]). In the most recently published GWAS of aggression with multiple observations in 87,485 children from ages 1.5–18 across multiple sites, instruments, and study designs, SNP heritability was reported to be 3.31% [118]. Though no genome-wide significant SNPs were found, three genes emerged as showing association with childhood aggression from gene-based analysis: *ST3GAL3* (*p* = 1.6e–6), *PCDH7* (*p* = 2.0e–6), and *IPO13* (*p* = 2.5e–6). The authors also reported significant genetic correlation between aggression and 36 phenotypes, including positive correlations between aggression and ADHD, smoking, major depressive disorder, and autism spectrum disorder, as well as negative correlations between aggression and age at smoking initiation, intelligence, and educational attainment [118].

### Mendelian randomization studies

One potentially powerful way in which genes have been used in the research literature is to clarify the causal mechanism between a predictor variable and outcome. This approach, known as Mendelian Randomization, uses genes as an instrumental variable, that is, a variable that predicts the predictor variable but not other confounding variables. Because genetic variants are inherited at random from the parents to their child, it can act as a quasi-randomized experiment.

Only one study was identified that used Mendelian Randomization to examine childhood aggression. Chao et al. [119] sought to examine the causal effect of alcohol consumption during adolescence and externalizing behaviors (including aggression; evaluated by Youth Self Report [120]) in 1608 Chinese adolescents. The Glu504Lys (rs671) polymorphism within the aldehyde dehydrogenase 2 family member-encoding *ALDH2* gene, having established effects on enzyme function [121–123] and consistent associations with alcohol use-related phenotypes [124, 125], was used as the instrumental variable. The results showed that decreased *ALDH2* function was significantly associated with lower alcohol use, and also with lower aggression problems. Alcohol use was found to be a significant mediator of the relationship between *ALDH2* and aggression, thus supporting the hypothesis that alcohol use causes adolescent aggression.

### Epigenetic studies

Our search resulted in two epigenetic studies. Provençal and colleagues [126] conducted a case-control study for 8 high-aggression case and 12 control participants and studied T cell DNA methylation using methylated DNA immunoprecipitation (MeDIP) followed by hybridization to microarrays. Their results reported that 227 and 171 distinct gene promoters were methylated significantly more in the control and high aggression group, respectively. From the differentially methylation genes, *AVPR1A*, *HTR1D* and *GRM5* were less methylated while *DRD1* and *SLC6A3* were more methylated in the high aggression group. More recently, Cecil and colleagues [127] demonstrated that there were seven differentially methylated sites across the genome in children who developed early onset conduct problems from an epigenome-wide association study (EWAS). Results of their follow-up studies with 15 candidate genes that were previously studied in relation to childhood aggression demonstrated that *MAOA, BDNF* and *FKBP5* were further associated with early onset of conduct problems in children [127].

## Discussion

**Fig. 2:**
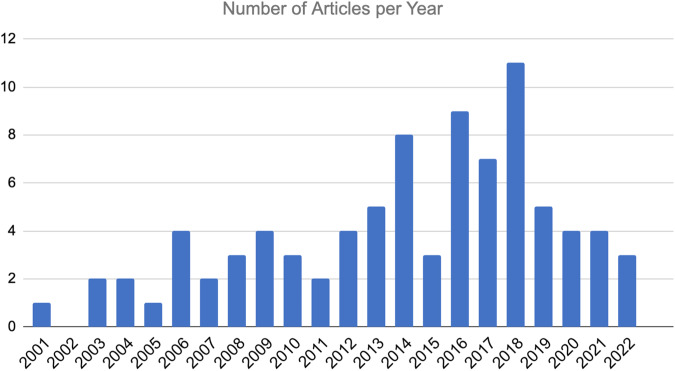
Number of childhood aggression studies. A histogram showing the number of childhood aggression genetic studies published per year.

To our knowledge, this is the first systematic review that specifically focuses on the genetics of childhood aggression. Overall, there is growing interest in this research area, as evidenced by the growing number of studies since 2001 (Fig. 2). Twin and pedigree studies support a prominent genetic component in liability for childhood aggression, which encourages further research to replicate and clarify findings from existing literature. The majority of gene association studies were candidate gene studies, which have focused on the *MAOA, DRD4* and *COMT* genes with mixed findings of their main effects. For the majority of candidate genes we reviewed, the positive findings (if any) have not been replicated in childhood aggression GWASs thus far [128]. It should be noted that many of the earlier childhood aggression candidate gene studies and GWASs were limited by insufficient sample sizes, lending itself to potential spurious relationship reportings and overestimation of effect sizes, a phenomenon known as the winner’s curse [129, 130]. Nonetheless, we found converging evidence for a role of *AVPR1A* in child aggression coming from genome-wide association [35], epigenomic [126], and candidate gene [101] studies. This warrants further investigation into the mechanism through which *AVPR1A* affects risk of child aggression and demonstrates that the use of diverse genetic study methodologies can facilitate genetic discoveries.

The conclusions from this review should be interpreted with the following considerations. Firstly, only studies that were in the English language were included, which may have biased the results to studies examining primarily European participants. Second, because our main focus for this systematic review was to shed light on the genetics of childhood aggression using only mesh terms of variants of the word aggression, studies that did not have direct assessments of childhood aggression or used only psychiatric diagnoses (e..g, ADHD, conduct disorder, oppositional defiant disorder) as proxies for aggressive behaviors would have been excluded. Furthermore, with null findings possibly not being reported and statistically significant findings tending to be published, publication bias is likely when drawing generalized conclusions from these published results [131].

Quality assessment of studies included in this review identified a number of areas where improvements will help advance the field of child aggression genetics. Sample size is a major limitation, with 74% of the studies being rated as moderate to serious in our quality assessment (Supplementary Table S1). Although it is more apparent in earlier candidate gene studies, it also remains a limiting factor in identifying genetic markers for child aggression GWASs. As many genetic studies also examined gene-gene and/or gene-environment interactions, even larger sample sizes are required. Another consideration is the definition and measurement of child aggression, for which 75% of the studies have been rated as moderate to critical (Table S1). The assessment methods of aggression varied substantially across studies in terms of tools and informants **(**Table 1**)**, which may have increased heterogeneity and limited comparability across studies. Another consideration is the variability in the inclusion of potential confounding factors as well as that of environmental factors being examined in interaction with genetic factors, for which 95% of the articles have been rated as moderate to serious (Table S1). There are numerous prenatal and postnatal environmental factors, such as socioeconomic status, childhood trauma, abuse and maltreatment, parenting styles, maternal sensitivity, prenatal stress, parental psychiatric disorders and alcohol use, that may influence the effects of genes on aggressive behaviors (reviewed in [132]). Studies using standardized measures of aggression and considering multiple environmental and confounding factors will help to disentangle the complexity surrounding child aggression.

Moreover, studies were limited with their participant selections where 92% of the studies has been rated as moderate to serious **(**Table S1**)**. While the majority of the studies included only participants of European ancestry (Table S2) in order to limit spurious findings due to population stratification, the results may not be generalizable to participants of other ancestries [63, 133]. More studies on participants of non-European ancestries are important in gaining additional insights into biological pathways for child aggression [134], as demonstrated in multi-ancestry GWASs of other phenotypes such as asthma [135] and rheumatoid arthritis [136]. Moreover, 16 studies included in this review only included male participants, while only one study included only female participants. Sex is a major factor that may modify the gene-behavior association. While males are three times more likely to exhibit aggressive behaviors than females due to both biological and cultural factors [137–139], the effects of genes on behavior may also be modified by sex-specific factors such as the levels of testosterone and Y-chromosome genes [140, 141]. Therefore more studies focusing on females are necessary to understand the genetics underlying female youth aggressive behaviors.

Furthermore, developmental age is another major factor that may change the effects of genes on childhood aggression [142] due to factors including the changing levels of gene expression, hormones, and enzymatic activity during development [142–144]. Study designs and data analyses that account for age and/or development in their study designs and data analyses, as age-stratified analyses [47, 67] and longitudinal assessments for changes in aggressive behavior and related factors may uncover novel associations and clarify mixed findings in the literature.

Lastly, it is important to note that GWAS does not directly interrogate other types of genetic variants, such as repeat polymorphisms (e.g., *MAOA*-uVTNR, 5-HTTLPR, *DRD4* exon III VNTR, *AVPR1A* RS1 and RS3). Examining the correlation between SNPs and these repeat polymorphisms will help in incorporating this type of polymorphisms in GWAS. Incorporation of rare variants, copy number variants, and other genetic variants besides SNPs and repeat polymorphisms in whole-genome analyses would likely help in explaining additional portions of the risk for child aggression and understanding the genetic architecture underlying child aggression [145–149]. Building consensus on the designation of risk vs. non-risk alleles, low vs. high activity genotypes, and short vs. long allele cutoffs in repeat polymorphisms will also facilitate the interpretation and generalizability of research findings across studies.

There are many future directions that can be followed from the results and limitations found from our systematic review of the literature. Most prominently, with candidate gene studies continuing to dominate the field of childhood aggression research, there is a greater need for more varied approaches, including epigenetic studies, gene expression studies, interrogation of rare [145] and/or more complex variants [148] in addition to SNPs, gene system studies, longitudinal studies that track changes in risk/ameliorating factors and aggression-related outcomes, as well as studies examining causal mechanisms related to aggressive behavior.

With the exception of ADHD and autism spectrum disorder, there is a paucity of well-powered GWASs in pediatric populations [150, 151], especially for aggressive behaviors and related phenotypes such as disruptive behavior disorders, conduct disorders, as well as externalizing and internalizing behaviors. There are a few studies that have investigated aggression-related phenotypes in the context of ADHD and other psychiatric disorders using summary data such as the Psychiatric Genetics Consortium, with one study noting an increased contribution of common genetic variants to ADHD with disruptive behavior disorder compared to ADHD without disruptive behavior disorder, with a portion of that increase attributed to genetic variants associated with aggression [152]. Genomic analyses of the genetic architectures of aggression and related phenotypes in youth as well as their co-occurrences will improve our understanding of the unique and shared genetic components across these phenotypes and across the lifespan [110, 152]. Therefore, future research is warranted focusing on the shared genetic architecture of aggression and the related phenotypes.

## Conclusion

Extreme and persistent childhood aggression continues to be a public health concern worldwide with potentially serious lifelong consequences to the perpetrator, the victim, and their loved ones, as well as incurring major costs to the society as a whole [153]. To devise effective early identification, intervention, and prevention strategies, an understanding of the biological mechanisms and environmental determinants of excessive childhood aggression is paramount. However, it is crucial to consider the factors such as sex, environment, development, and ethnicity when analyzing the effects of genes on child aggression. Although we found that the quality of the reviewed studies improved over time, the overall risk of bias for 95% of current evidence were rated as moderate to serious (Table S1). Improvement to the research design including larger sample size and standardized, reliable assessment of aggressive behavior, as well as triangulation of research evidence using diverse genetic research methodologies, will facilitate the advancement of genetic research in childhood aggression.

### Supplementary information

Supplementary Materials
