# Supplementary material for: Genetics of child aggression, a systematic review
Source: Transl Psychiatry. 2024 Jun 11;14:252. doi: 10.1038/s41398-024-02870-7 (PMC11167064; doi:10.1038/s41398-024-02870-7)
Supplement: Supplementary file 1 — Supplementary Materials [file 41398_2024_2870_MOESM1_ESM.docx]

Table S1. Quality Assessment of Reviewed Articles

| **Year** | **Study ID** | **Title** | **Sample Size** | **Confounding** | **Participant Selection** | **Measurement of Outcomes** | **Selection of reported results** | **Overall Risk of Bias** | **General Comments** |
| --- | --- | --- | --- | --- | --- | --- | --- | --- | --- |
| 2001 | Twitchell 2001 | Serotonin transporter promoter polymorphism genotype is associated with behavioral disinhibition and negative affect in children of alcoholics. | Serious Risk | Moderate Risk | Moderate Risk | Moderate Risk | Moderate Risk | Moderate Risk |  |
| 2003 | Cadoret 2003 | Associations of the serotonin transporter promoter polymorphism with aggressivity, attention deficit, and conduct disorder in an adoptee population. | Serious Risk | Moderate Risk | Serious Risk | Serious Risk | Moderate Risk | Serious Risk |  |
| 2003 | Lawson 2003 | Association analysis of monoamine oxidase A and attention deficit hyperactivity disorder. | Serious Risk | Moderate Risk | Serious Risk | Serious Risk | Moderate Risk | Serious Risk | How ADHD/CD is diagnosed unclear |
| 2004 | Beitchman 2004 | MAOA and persistent, pervasive childhood aggression. | Serious Risk | Serious Risk | Moderate Risk | Serious Risk | Moderate Risk | Serious Risk |  |
| 2004 | Davidge 2004 | Association of the serotonin transporter and 5HT1Dbeta receptor genes with extreme, persistent and pervasive aggressive behaviour in children. | Serious Risk | Serious Risk | Moderate Risk | Low Risk | Moderate Risk | Moderate Risk |  |
| 2005 | Bearden 2005 | Effects of COMT genotype on behavioral symptomatology in the 22q11.2 Deletion Syndrome | Serious Risk | Serious Risk | Moderate Risk | Moderate Risk | Moderate Risk | Serious Risk |  |
| 2006 | Bakermans-Kranenburg 2006 | Gene-environment interaction of the dopamine D4 receptor (DRD4) and observed maternal insensitivity predicting externalizing behavior in preschoolers. | Critical | Serious Risk | Moderate Risk | Moderate Risk | Moderate Risk | Serious Risk | Ancestry not controlled for |
| 2006 | DeYoung 2006 | The dopamine D4 receptor gene and moderation of the association between externalizing behavior and IQ. | Moderate Risk | Moderate Risk | Moderate Risk | Moderate Risk | Moderate Risk | Moderate Risk |  |
| 2006 | Haberstick 2006 | Family-based association test of the 5HTTLPR and aggressive behavior in a general population sample of children. | Low Risk | Moderate Risk | Moderate Risk | Low Risk | Moderate Risk | Moderate Risk |  |
| 2006 | Beitchman 2006 | Serotonin transporter polymorphisms and persistent, pervasive childhood aggression. | Serious Risk | Serious Risk | Moderate Risk | Moderate Risk | Moderate Risk | Serious Risk |  |
| 2007 | Nobile 2007 | Socioeconomic status mediates the genetic contribution of the dopamine receptor D4 and serotonin transporter linked promoter region polymorphisms to externalization in preadolescence. | Moderate Risk | Moderate Risk | Moderate Risk | Moderate Risk | Moderate Risk | Moderate Risk |  |
| 2007 | Feinberg 2007 | Parenting and adolescent antisocial behavior and depression: evidence of genotype x parenting environment interaction. | Low Risk | Moderate Risk | Low Risk | Moderate Risk | Moderate Risk | Moderate Risk |  |
| 2008 | Oades 2008 | The influence of serotonin- and other genes on impulsive behavioral aggression and cognitive impulsivity in children with attention-deficit/hyperactivity disorder (ADHD): Findings from a family-based association test (FBAT) analysis. | Low Risk | Moderate Risk | Moderate Risk | Moderate Risk | Moderate Risk | Moderate Risk |  |
| 2008 | Caspi 2008 | A replicated molecular genetic basis for subtyping antisocial behavior in children with attention-deficit/hyperactivity disorder. | Low Risk | Moderate Risk | Moderate Risk | Moderate Risk | Moderate Risk | Moderate Risk |  |
| 2008 | Shleptsova 2008 | Role of renin-angiotensin system in the formation of emotional state in humans. | Moderate Risk | Moderate Risk | Serious Risk | Moderate Risk | Moderate Risk | Moderate Risk |  |
| 2009 | Hohmann 2009 | Evidence for epistasis between the 5-HTTLPR and the dopamine D4 receptor polymorphisms in externalizing behavior among 15-year-olds. | Moderate Risk | Serious Risk | Moderate Risk | Low Risk | Moderate Risk | Moderate Risk |  |
| 2009 | Weder 2009 | MAOA genotype, maltreatment, and aggressive behavior: the changing impact of genotype at varying levels of trauma. | Serious Risk | Moderate Risk | Moderate Risk | Moderate Risk | Moderate Risk | Moderate Risk |  |
| 2009 | DiLalla 2009 | Genetic and gene-environment interaction effects on preschoolers' social behaviors. | Critical | Moderate Risk | Moderate Risk | Low Risk | Moderate Risk | Serious Risk |  |
| 2009 | Sysoeva 2009 | Aggression and 5HTT polymorphism in females: study of synchronized swimming and control groups. | Serious Risk | Moderate Risk | Moderate Risk | Moderate Risk | Moderate Risk | Moderate Risk |  |
| 2010 | Kiive 2010 | Effect of alpha2A-adrenoceptor C-1291G genotype and maltreatment on hyperactivity and inattention in adolescents. | Moderate Risk | Moderate Risk | Moderate Risk | Moderate Risk | Moderate Risk | Moderate Risk |  |
| 2010 | Edwards 2010 | MAOA-uVNTR and early physical discipline interact to influence delinquent behavior. | Serious Risk | Moderate Risk | Moderate Risk | Low Risk | Moderate Risk | Moderate Risk |  |
| 2010 | Albaugh 2010 | COMT Val158Met genotype as a risk factor for problem behaviors in youth. | Serious Risk | Moderate Risk | Serious Risk | Moderate Risk | Low Risk | Serious Risk | Ancestry unclear |
| 2011 | Dick 2011 | CHRM2, parental monitoring, and adolescent externalizing behavior: evidence for gene-environment interaction. | Moderate Risk | Moderate Risk | Moderate Risk | Low Risk | Moderate Risk | Moderate Risk |  |
| 2011 | Mick 2011 | Genome-wide association study of the child behavior checklist dysregulation profile. | Critical | Moderate Risk | Moderate Risk | Moderate Risk | Moderate Risk | Serious Risk | Within-family design |
| 2012 | Zai 2012 | Possible genetic association between vasopressin receptor 1B and child aggression | Moderate Risk | Moderate Risk | Serious Risk | Low Risk | Moderate Risk | Moderate Risk |  |
| 2012 | Malik 2012 | The role of oxytocin and oxytocin receptor gene variants in childhood-onset aggression. | Serious Risk | Moderate Risk | Serious Risk | Low Risk | Moderate Risk | Moderate Risk |  |
| 2012 | Zai 2012 | Dopaminergic system genes in childhood aggression: possible role for DRD2. | Moderate Risk | Moderate Risk | Moderate Risk | Low Risk | Moderate Risk | Moderate Risk |  |
| 2012 | Beitchman 2012 | Childhood aggression, callous-unemotional traits and oxytocin genes. | Serious Risk | Serious Risk | Moderate Risk | Serious Risk | Moderate Risk | Serious Risk |  |
| 2013 | Pingault 2013 | Age-dependent effect of the MAOA gene on childhood physical aggression. | Moderate Risk | Moderate Risk | Moderate Risk | Moderate Risk | Moderate Risk | Moderate Risk |  |
| 2013 | Pickles 2013 | Evidence for interplay between genes and parenting on infant temperament in the first year of life: monoamine oxidase A polymorphism moderates effects of maternal sensitivity on infant anger proneness. | Moderate Risk | Moderate Risk | Moderate Risk | Moderate Risk | Moderate Risk | Moderate Risk |  |
| 2013 | Hirata 2013 | Study of the catechol-o-methyltransferase (COMT) gene with high aggression in children. | Moderate Risk | Moderate Risk | Serious Risk | Low Risk | Low Risk | Moderate Risk |  |
| 2013 | Hamshere 2013 | High loading of polygenic risk for ADHD in children with comorbid aggression. | Low Risk | Moderate Risk | Serious Risk | Serious Risk | Moderate Risk | Moderate Risk |  |
| 2013 | Hakulinen 2013 | Serotonin receptor 1B genotype and hostility, anger and aggressive behavior through the lifespan: the Young Finns study. | Low Risk | Moderate Risk | Moderate Risk | Serious Risk | Moderate Risk | Moderate Risk |  |
| 2014 | Zohsel 2014 | Mothers' prenatal stress and their children's antisocial outcomes--a moderating role for the dopamine D4 receptor (DRD4) gene. | Moderate Risk | Low Risk | Moderate Risk | Moderate Risk | Moderate Risk | Moderate Risk |  |
| 2014 | Villafuerte 2014 | Genetic variation in GABRA2 moderates peer influence on externalizing behavior in adolescents. | Moderate Risk | Moderate Risk | Moderate Risk | Moderate Risk | Moderate Risk | Moderate Risk |  |
| 2014 | Kiive 2014 | Mitigating aggressiveness through education? The monoamine oxidase A genotype and mental health in general population. | Moderate Risk | Moderate Risk | Moderate Risk | Moderate Risk | Moderate Risk | Moderate Risk |  |
| 2014 | Lacourse 2014 | A longitudinal twin study of physical aggression during early childhood: evidence for a developmentally dynamic genome. | Moderate Risk | Moderate Risk | Moderate Risk | Serious Risk | Moderate Risk | Moderate Risk |  |
| 2014 | Malik 2014 | The role of genetic variants in genes regulating the oxytocin-vasopressin neurohumoral system in childhood-onset aggression. | Moderate Risk | Moderate Risk | Moderate Risk | Low Risk | Moderate Risk | Moderate Risk |  |
| 2014 | Hygen 2014 | Catechol-O-methyltransferase Val158Met genotype moderates the effect of disorganized attachment on social development in young children. | Low Risk | Moderate Risk | Moderate Risk | Moderate Risk | Moderate Risk | Moderate Risk |  |
| 2014 | Provençal 2014 | Association of childhood chronic physical aggression with a DNA methylation signature in adult human T cells. | Serious Risk | Serious Risk | Serious Risk | Serious Risk | Moderate Risk | Serious Risk |  |
| 2014 | Farbiash 2014 | Prediction of preschool aggression from DRD4 risk, parental ADHD symptoms, and home chaos. | Serious Risk | Moderate Risk | Moderate Risk | Moderate Risk | Moderate Risk | Moderate Risk |  |
| 2015 | Salvatore 2015 | Intergenerational continuity in parents' and adolescents' externalizing problems: The role of life events and their interaction with GABRA2. | Moderate Risk | Moderate Risk | Low Risk | Moderate Risk | Moderate Risk | Moderate Risk |  |
| 2015 | Thibodeau 2015 | Child maltreatment, impulsivity, and antisocial behavior in African American children: Moderation effects from a cumulative dopaminergic gene index. | Low Risk | Moderate Risk | Moderate Risk | Moderate Risk | Moderate Risk | Moderate Risk |  |
| 2015 | Hygen 2015 | Child exposure to serious life events, COMT, and aggression: Testing differential susceptibility theory. | Low Risk | Moderate Risk | Moderate Risk | Moderate Risk | Moderate Risk | Moderate Risk |  |
| 2016 | Trucco 2016 | Susceptibility effects of GABA receptor subunit alpha-2 (GABRA2) variants and parental monitoring on externalizing behavior trajectories: Risk and protection conveyed by the minor allele. | Moderate Risk | Moderate Risk | Moderate Risk | Moderate Risk | Moderate Risk | Moderate Risk |  |
| 2016 | Bryushkova 2016 | FKBP5 interacts with maltreatment in children with extreme, pervasive, and persistent aggression. | Moderate Risk | Moderate Risk | Serious Risk | Moderate Risk | Moderate Risk | Moderate Risk |  |
| 2016 | Hirata 2016 | Possible association between the prolactin receptor gene and callous-unemotional traits among aggressive children. | Moderate Risk | Moderate Risk | Serious Risk | Low Risk | Low Risk | Serious Risk |  |
| 2016 | Zhang 2016 | The interactive effect of the MAOA-VNTR genotype and childhood abuse on aggressive behaviors in Chinese male adolescents. | Moderate Risk | Moderate Risk | Low Risk | Moderate Risk | Low Risk | Moderate Risk | No history of psychiatric or neurological problems according to self-reports; SCID was administered |
| 2016 | Ma 2016 | Electrophysiological responses of feedback processing are modulated by MAOA genotype in healthy male adolescents. | Serious Risk | Moderate Risk | Moderate Risk | Moderate Risk | Moderate Risk | Moderate Risk |  |
| 2016 | vanGoozen 2016 | Identifying mechanisms that underlie links between COMT genotype and aggression in male adolescents with ADHD. | Moderate Risk | Moderate Risk | Moderate Risk | Moderate Risk | Moderate Risk | Moderate Risk |  |
| 2016 | Brevik 2016 | Genome-wide analyses of aggressiveness in attention-deficit hyperactivity disorder. | Low Risk | Moderate Risk | Moderate Risk | Moderate Risk | Moderate Risk | Moderate Risk |  |
| 2016 | Zhang 2016 | Monoamine Oxidase A (MAOA) and Catechol-O-Methyltransferase (COMT) Gene Polymorphisms Interact with Maternal Parenting in Association with Adolescent Reactive Aggression but not Proactive Aggression: Evidence of Differential Susceptibility. | Low Risk | Moderate Risk | Moderate Risk | Moderate Risk | Moderate Risk | Moderate Risk |  |
| 2016 | Pappa 2016 | A genome-wide approach to children's aggressive behavior: The EAGLE consortium. | Low Risk | Low Risk | Low Risk | Moderate Risk | Moderate Risk | Moderate Risk |  |
| 2017 | Zhang 2017 | Gene-gene-environment interactions of serotonin transporter, monoamine oxidase a and childhood maltreatment predict aggressive behavior in Chinese adolescents. | Low Risk | Moderate Risk | Moderate Risk | Moderate Risk | Moderate Risk | Moderate Risk |  |
| 2017 | Galán 2017 | The interaction between monoamine oxidase A and punitive discipline in the development of antisocial behavior: Mediation by maladaptive social information processing. | Serious Risk | Serious Risk | Moderate Risk | Moderate Risk | Moderate Risk | Moderate Risk |  |
| 2017 | Liu 2017 | Association of Y-linked variants with impulsivity and aggression in boys with attention-deficit/hyperactivity disorder of Chinese Han descent. | Moderate Risk | Moderate Risk | Moderate Risk | Moderate Risk | Moderate Risk | Moderate Risk |  |
| 2017 | Laas 2017 | Nice guys: Homozygocity for the TPH2 -703G/T (rs4570625) minor allele promotes low aggressiveness and low anxiety. | Low Risk | Moderate Risk | Moderate Risk | Moderate Risk | Serious Risk | Moderate Risk |  |
| 2017 | Chao 2017 | The Causal Role of Alcohol Use in Adolescent Externalizing and Internalizing Problems: A Mendelian Randomization Study. | Moderate Risk | Moderate Risk | Moderate Risk | Moderate Risk | Moderate Risk | Moderate Risk |  |
| 2017 | Gillentine 2017 | CHRNA7 Deletions are Enriched in Risperidone-Treated Children and Adolescents. | Moderate Risk | Serious Risk | Moderate Risk | Moderate Risk | Moderate Risk | Moderate Risk | Ancestry unclear |
| 2017 | Kiive 2017 | Stressful life events increase aggression and alcohol use in young carriers of the GABRA2 rs279826/rs279858 A-allele. | Moderate Risk | Moderate Risk | Moderate Risk | Moderate Risk | Moderate Risk | Moderate Risk |  |
| 2018 | CecilCAM 2018 | Neonatal DNA methylation and early-onset conduct problems: A genome-wide, prospective study. | Moderate Risk | Moderate Risk | Moderate Risk | Moderate Risk | Moderate Risk | Moderate Risk |  |
| 2018 | vanDonkelaar 2018 | Pleiotropic contribution of MECOM and AVPR1A to aggression and subcortical brain volumes. | Low Risk | Moderate Risk | Moderate Risk | Low Risk | Moderate Risk | Low Risk |  |
| 2018 | Wang 2018 | Interacting effect of catechol-O-methyltransferase (COMT) and monoamine oxidase a (MAOA) gene polymorphisms, and stressful life events on aggressive behavior in Chinese male adolescents. | Low Risk | Moderate Risk | Moderate Risk | Low Risk | Moderate Risk | Low Risk |  |
| 2018 | Glenn 2018 | Oxytocin Receptor Gene Variant Interacts with Intervention Delivery Format in Predicting Intervention Outcomes for Youth with Conduct Problems. | Moderate Risk | Moderate Risk | Moderate Risk | Moderate Risk | Moderate Risk | Moderate Risk |  |
| 2018 | Paes 2018 | Association between serotonin 2C receptor gene (HTR2C) polymorphisms and psychopathological symptoms in children and adolescents. | Serious Risk | Serious Risk | Moderate Risk | Moderate Risk | Moderate Risk | Moderate Risk |  |
| 2018 | Shao 2018 | Effect of the interaction between oxytocin receptor gene polymorphism (rs53576) and stressful life events on aggression in Chinese Han adolescents. | Serious Risk | Moderate Risk | Moderate Risk | Moderate Risk | Moderate Risk | Moderate Risk |  |
| 2018 | Musci 2018 | Evaluating the genetic susceptibility to peer reported bullying behaviors. | Serious Risk | Moderate Risk | Moderate Risk | Serious Risk | Moderate Risk | Moderate Risk |  |
| 2018 | VanHulle 2018 | Sex Differences in the Genetic and Environmental Influences on Self-Reported Non-aggressive and Aggressive Conduct Disorder Symptoms in Early and Middle Adolescence. | Moderate Risk | Moderate Risk | Moderate Risk | Moderate Risk | Moderate Risk | Moderate Risk |  |
| 2018 | Wang 2018 | Serotonin functioning and adolescents' alcohol use: A genetically informed study examining mechanisms of risk. | Moderate Risk | Moderate Risk | Moderate Risk | Moderate Risk | Moderate Risk | Moderate Risk |  |
| 2018 | Jamnik 2018 | A Multimethodological Study of Preschoolers' Preferences for Aggressive Television and Video Games. | Moderate Risk | Moderate Risk | Moderate Risk | Low Risk | Moderate Risk | Moderate Risk |  |
| 2018 | Achterberg 2018 | Heritability of aggression following social evaluation in middle childhood: An fMRI study. | Moderate Risk | Moderate Risk | Moderate Risk | Moderate Risk | Moderate Risk | Moderate Risk | Ancestry not controlled for |
| 2019 | Elam 2019 | Gene set enrichment analysis to create polygenic scores: a developmental examination of aggression. | Moderate Risk | Low Risk | Moderate Risk | Moderate Risk | Moderate Risk | Moderate Risk |  |
| 2019 | Musci 2019 | Violence exposure in an urban city: A GxE interaction with aggressive and impulsive behaviors. | Moderate Risk | Moderate Risk | Moderate Risk | Moderate Risk | Moderate Risk | Moderate Risk |  |
| 2019 | Sener 2019 | Altered Global mRNA Expressions of Pain and Aggression Related Genes in the Blood of Children with Autism Spectrum Disorders. | Serious Risk | Moderate Risk | Moderate Risk | Critical | Serious Risk | Serious Risk |  |
| 2019 | Slawinski 2019 | The etiology of social aggression: a nuclear twin family study. | Low Risk | Low Risk | Low Risk | Moderate Risk | Low Risk | Low Risk |  |
| 2019 | Vestlund 2019 | Ghrelin and aggressive behaviours-Evidence from preclinical and human genetic studies. | Low Risk | Moderate Risk | Moderate Risk | Moderate Risk | Moderate Risk | Moderate Risk |  |
| 2020 | Wang 2020 | Evidence for two genetically distinct pathways to co-occurring internalizing and externalizing problems in adolescence characterized by negative affectivity or behavioral inhibition. | Moderate Risk | Moderate Risk | Moderate Risk | Moderate Risk | Moderate Risk | Moderate Risk |  |
| 2020 | Liu 2020 | Association of Corticotropin-Releasing Hormone Receptor-1 Gene Polymorphisms and Personality Traits with Violent Aggression in Male Adolescents. | Moderate Risk | Serious Risk | Moderate Risk | Moderate Risk | Moderate Risk | Moderate Risk |  |
| 2020 | Letourneau 2020 | Parenting Interacts With Plasticity Genes in Predicting Behavioral Outcomes in Preschoolers. | Serious Risk | Moderate Risk | Moderate Risk | Moderate Risk | Moderate Risk | Moderate Risk |  |
| 2020 | Orri 2020 | Contribution of genes and environment to the longitudinal association between childhood impulsive-aggression and suicidality in adolescence. | Low Risk | Low Risk | Moderate Risk | Moderate Risk | Moderate Risk | Moderate Risk |  |
| 2021 | Vaht 2021 | Variation rs6971 in the translocator protein gene (TSPO) is associated with aggressiveness and impulsivity but not with anxiety in a population-representative sample of young adults. | Moderate Risk | Serious Risk | Moderate Risk | Moderate Risk | Moderate Risk | Moderate Risk |  |
| 2021 | Womack 2021 | Genetic Moderation of the Association Between Early Family Instability and Trajectories of Aggressive Behaviors from Middle Childhood to Adolescence. | Moderate Risk | Moderate Risk | Moderate Risk | Low Risk | Moderate Risk | Moderate Risk |  |
| 2021 | Vollebregt 2021 | Evidence for association of vasopressin receptor 1A promoter region repeat with childhood onset aggression. | Moderate Risk | Moderate Risk | Low Risk | Low Risk | Low Risk | Moderate Risk |  |
| 2021 | Ip 2021 | Genetic association study of childhood aggression across raters, instruments, and age. | Low Risk | Moderate Risk | Low Risk | Low Risk | Low Risk | Low Risk |  |
| 2022 | Kant 2022 | Association of the MAOA-uVNTR polymorphism with psychopathic traits may change from childhood to adolescence. | Moderate Risk | Moderate Risk | Moderate Risk | Low Risk | Moderate Risk | Moderate Risk |  |
| 2022 | Kant 2022 | COMT Val/Met and Psychopathic Traits in Children and Adolescents: A Systematic Review and New Evidence of a Developmental Trajectory toward Psychopathy. | Moderate Risk | Moderate Risk | Moderate Risk | Low Risk | Low Risk | Moderate Risk |  |
| 2022 | NedicErjavec 2022 | Serotonin 5-HT(2A) receptor polymorphisms are associated with irritability and aggression in conduct disorder. | Serious Risk | Moderate Risk | Moderate Risk | Low Risk | Moderate Risk | Moderate Risk |  |

Table S2. Number of Articles by Ethnicity of the Majority of Study Participants

| **Major Ethnicity** | **Number of Studies** |  |
| --- | --- | --- |
| European | 61 |  |
| Chinese | 9 |  |
| African American | 5 |  |
| Other | 12 |  |
| **Total** | **87** |  |
